# Towards Microalgal Biorefinery: Multiproduct Fractionation of *Phaeodactylum tricornutum* by Liquid–Liquid Techniques

**DOI:** 10.3390/md24070242

**Published:** 2026-07-09

**Authors:** Kolos Makay, Claudia Grewe

**Affiliations:** Doctoral Center Life Sciences, Research Center and Graduate School of Anhalt University of Applied Sciences, Bernburger Str. 55, 06366 Köthen, Germany; kolos.makay@hs-anhalt.de

**Keywords:** fucoxanthin, EPA, solid–liquid–liquid extraction, centrifugal partition chromatography, biorefinery, *Phaeodactylum tricornutum*

## Abstract

*Phaeodactylum tricornutum* is a promising biorefinery feedstock because it contains high-value compounds such as fucoxanthin and eicosapentaenoic acid (EPA), alongside other pigments, proteins, carbohydrates, and polyphenolics. However, downstream processing often targets single compounds, leaving possible co-products underutilised, thus limiting biomass valorisation. This study developed a multiproduct workflow for wet, disrupted *P. tricornutum* biomass by coupling solid–liquid–liquid extraction (SLLE) with centrifugal partition chromatography (CPC). The SLLE step used an ethyl acetate/n-butanol/water solvent system (3:2:5, *v*/*v*/*v*) and was optimised with respect to biomass loading and extraction time, yielding lipophilic, aqueous, interfacial, and insoluble primary fractions. Biomass content was the dominant factor governing partitioning into these fractions and target-compound recovery, whereas extraction time had a secondary influence. Under process-oriented optimised conditions of 1.3 h and 4.25% biomass content, fucoxanthin and EPA recoveries reached 91.3% and 70%, respectively. The lipophilic fraction was refined further by two-stage CPC, yielding high-purity fucoxanthin (99.4 ± 1.0%) and EPA-enriched glycerolipids (up to 99.9 ± 0.5%). Additionally, ten further fractions were obtained, including carotenoid-containing, chlorophyll, polyphenolic, protein-rich, and carbohydrate-rich fractions in the whole process. Overall, this twelve-fraction workflow supports the transition toward a scalable *P. tricornutum* biorefinery and provides a basis for assessing transferability to other microalgae.

## 1. Introduction

*Phaeodactylum tricornutum* (Bacillariophyceae) is a marine pennate diatom widely used as a model microalgae for molecular and biotechnological research [1,2]. The unicellular pleomorphic microalga forms fusiform, oval, and triradiate cells [3] that are of commercial interest owing to their high-value compounds [4]. These include the carotenoid fucoxanthin (~10–60 mg g^−1^ dry weight (DW)) [5] and the omega-3 long-chain polyunsaturated fatty acid (n-3 LC-PUFA) eicosapentaenoic acid (EPA; ~30–50 mg g^−1^ DW) [6], both associated with recognised bioactivities (e.g., antioxidant, anti-inflammatory, cardioprotective effects) [7,8]. Additional valuable constituents include β-glucans (~317 mg g^−1^ DW) [9], proteins (~350–420 mg g^−1^ DW) [10] and polyphenols (~2.9 mg gallic acid equivalent (GAE) g^−1^ DW) [11]. The biochemical profile of *P. tricornutum* can be influenced by cultivation conditions and medium composition, including light regime, temperature, salinity, carbon supply, and nutrient availability, which can shift cellular metabolism toward different target metabolites [12,13]. This compositional diversity and plasticity make *P. tricornutum* well suited to biorefinery concepts, enabling the recovery of a primary high-value product together with multiple valuable co-products [14,15].

However, the commercial exploitation of *P. tricornutum* remains limited [6]. Conventional downstream processing (DSP) routes typically involve harvesting followed by drying and supercritical CO_2_ extraction to recover lipophilic compounds such as fucoxanthin and EPA [16,17]. Although this process route enables the recovery of lipophilic products, it also results in a chemically complex, low-purity oleoresin with non-targeted co-extracted products. In addition, a substantial residual biomass fraction remains after supercritical CO_2_ extraction, retaining valuable constituents. Recovering the remaining compounds requires further processing steps, such as aqueous extraction [16,18]. Such sequential processing can increase overall biomass utilisation; however, it also adds unit operations and process complexity, as reflected in proposed cascading microalgal biorefinery schemes [15,19,20]. Accordingly, DSP is widely regarded as a major bottleneck in microalgal biotechnology. However, this limitation may not arise from DSP requirements per se, but rather from the way DSP workflows are currently conceptualised [21,22].

Thus, for biorefinery applications, DSP may be approached as an integrated fractionation strategy in which each unit operation further separates biomass-derived material into distinct valorisable streams, thereby broadening the range of recoverable products and co-products [23,24,25]. In microalgal processing, however, this objective must be reconciled with unavoidable preparatory operations. As microalgal suspensions are highly dilute (typically ~0.05–0.5% *w*/*w* DW), biomass concentration is required prior to further processing. Furthermore, cell disruption is necessary, as many target metabolites, such as fucoxanthin and EPA, are located intracellularly at the organelle level [26,27,28]. Together, these initial DSP operations convert the dilute culture into concentrated, disrupted biomass, which can then be processed through successive unit operations to recover a primary product while further separating biomass-derived material into additional valorisable product and co-product streams.

At this stage, solid–liquid–liquid extraction (SLLE), using aqueous–organic solvent systems, enables the simultaneous partitioning of chemically distinct metabolite classes into separate product streams, resulting in lipophilic, aqueous, and solid fractions. Although SLLE enables the generation of multiple fractions in a single step, the fractions remain chemically complex mixtures rather than discrete, well-defined products. Thus, further resolution of the lipid-enriched fraction is required to obtain fucoxanthin- and EPA-enriched fractions [29,30,31]. Rather than introducing additional DSP steps targeting individual compounds, this refinement can be achieved within the same liquid–liquid separation framework. In this context, liquid–liquid chromatography (LLC), particularly centrifugal partition chromatography (CPC), represents a suitable extension of SLLE-based fractionation. Based on repeated partitioning of solutes between two immiscible liquid phases, CPC enables the progressive resolution of complex extracts into compositionally distinct fractions. As no solid stationary phase is employed, irreversible adsorption is avoided, enabling high recoveries (>90%) and purities of enriched fractions typically in the range of 80–95%, while maintaining scalability from gram-scale separations to kilogram-scale throughput of crude extract per day [32,33]. Despite these advantages, the application of CPC and related support-free LLC techniques in microalgal processing has so far largely focused on the isolation of individual target compounds, such as fucoxanthin [34,35,36], lutein [37,38], or astaxanthin mono- and diesters [39,40].

**Figure 1:**
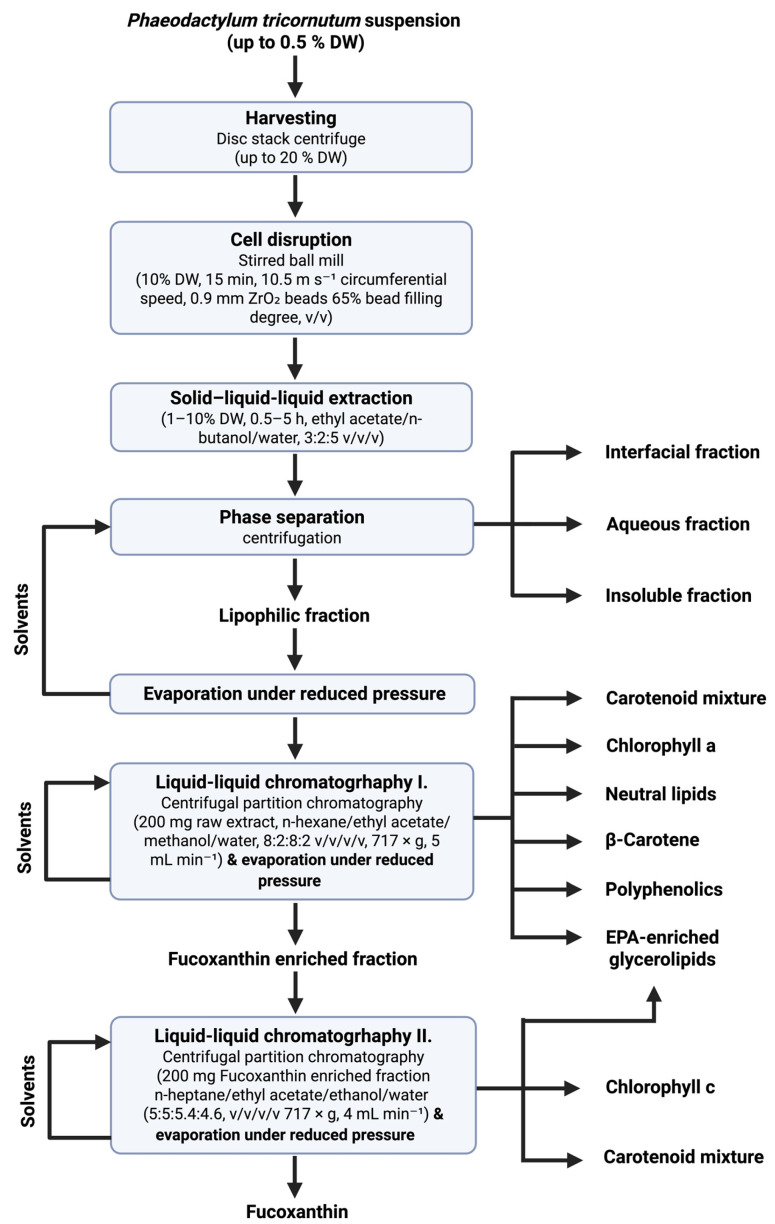
Process flow diagram for the enhanced valorisation of *Phaeodactylum tricornutum* biomass, including biomass harvesting, cell disintegration, solid–liquid–liquid extraction, phase separation, liquid–liquid chromatography, solvent recycling, and recovery of fucoxanthin as the main product together with the generated co-products. DW: Dry weight.

Therefore, this study aimed to develop a DSP concept coupling SLLE and LLC for the multiproduct fractionation of concentrated, disrupted *P. tricornutum* biomass, as outlined in Figure 1. Fucoxanthin was selected as the primary value-driving target compound, while EPA-containing lipid fractions were considered additional major product targets. Where feasible, we further aimed to design the process so that additional co-product streams could also be recovered (Figure 1). To this end, we propose a workflow in which SLLE serves as an early capture and pre-structuring step, followed by CPC-based refinement of the lipophilic extract. Overall, this study aimed to establish an integrated SLLE–CPC fractionation strategy that prioritises fucoxanthin recovery while enabling the parallel recovery of EPA-containing lipid fractions and other potential co-products, thereby supporting the broader valorisation of *P. tricornutum*.

## 2. Results and Discussion

### 2.1. Solid–Liquid–Liquid Extraction

#### 2.1.1. Effect of Extraction Time and Biomass Content

The biphasic ethyl acetate/n-butanol/water system (3:2:5, *v*/*v*/*v*) was investigated for the SLLE of *P. tricornutum*, focusing on the phase distribution and recovery of lipophilic and hydrophilic product streams. The selection of this solvent system was guided by the requirement for a water-containing biphasic extraction system capable of simultaneously extracting and partitioning compounds spanning a broad polarity range between the aqueous phase and the organic solvent-rich phase. Accordingly, the organic solvents were required to exhibit limited miscibility with water, a reliable phase-forming behaviour, and complementary solvation properties. Ethyl acetate and n-butanol were selected because even though they possess comparable intermediate polarity, expressed by Snyder polarity index values (P′ = 4.4 for ethyl acetate and P′ = 3.9 for n-butanol) [41], they differ in their hydrogen-bonding characteristics. Ethyl acetate is an aprotic solvent that primarily acts as a hydrogen-bond acceptor, whereas n-butanol is a protic solvent with both hydrogen-bond donor and acceptor capabilities [42]. The 3:2 ethyl acetate/n-butanol ratio was therefore selected to maintain ethyl acetate as the major organic component, supporting the recovery of lipophilic constituents, while incorporating a substantial n-butanol fraction to broaden the hydrogen-bonding capacity and solvation characteristics of the solvent-rich phase. This composition was considered a practical compromise between an ethyl acetate-dominated solvent phase and an alcohol-enriched solvent phase, thereby supporting the extraction of highly lipophilic and more polar/amphiphilic constituents. Furthermore, both ethyl acetate and n-butanol are classified as recommended solvents in the CHEM21 solvent selection guide [43], supporting their selection from a green chemistry perspective (health, environment and safety). The addition of five-volume parts of water provided an equal nominal aqueous-to-organic solvent ratio, enabling evaluation of the distribution of hydrophilic and lipophilic product streams under defined SLLE conditions.

**Figure 2:**
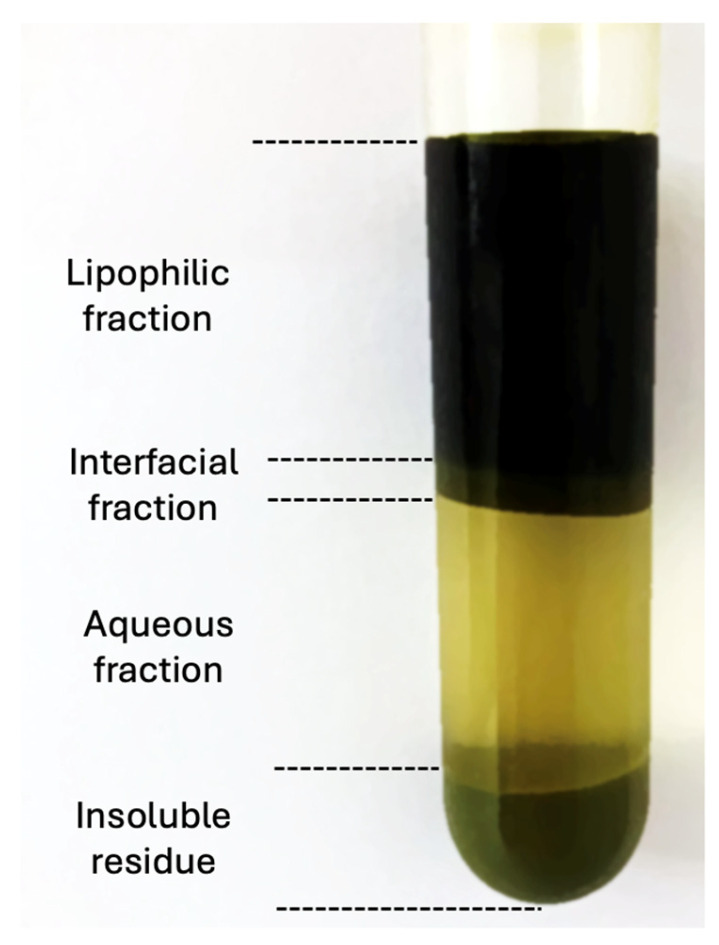
Phase distribution of fractions obtained from the solid-liquid-liquid extraction of disintegrated *Phaeodactylum tricornutum* (disintegration degree > 95%). Extraction was performed using an ethyl acetate/n-butanol/water (3:2:5, *v*/*v*/*v*) solvent system under optimised conditions (extraction time: 1.3 h; biomass loading: 4.25% *w*/*w*, dry weight basis). Phase separation was achieved by centrifugation at 4643× *g* for 2 h, yielding, from top to bottom, distinct lipophilic, interfacial, aqueous fractions, and an insoluble residue.

Particular emphasis was placed on fucoxanthin and EPA recovery in the lipophilic fraction, while the aqueous fraction was considered a co-product stream containing water-soluble constituents. Extraction time (0.5–5.0 h) and DW content (1–10%, *w*/*w*), reflecting the solvent-to-biomass ratio (*v*/*w*), were varied as continuous factors at 25 °C. After extraction and centrifugation, the system reproducibly separated into four layers: an ethyl acetate–n-butanol-rich organic phase, an interfacial layer, an aqueous phase, and a bottom pellet mainly composed of cell debris (Figure 2). Comparable multi-layer phase behaviour has been reported for *Nannochloropsis* sp. in an immiscible water–n-hexane system, where organic, emulsion, aqueous, and debris-rich layers were formed [23].

**Figure 3:**
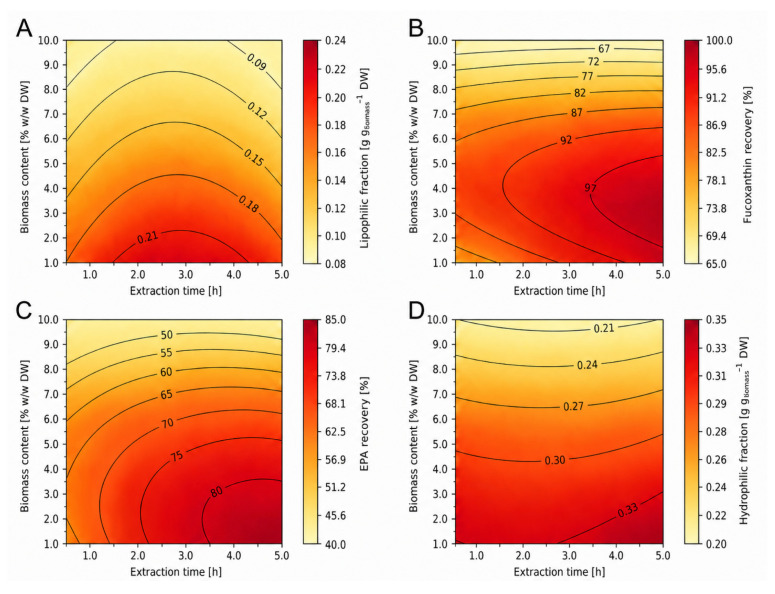
Response surface plots illustrating the influence of extraction time and biomass content (dry-weight (DW) basis) on (**A**) lipophilic fraction yield (mg g_biomass_^−1^ DW), (**B**) fucoxanthin recovery (%), (**C**) eicosapentaenoic acid (EPA) recovery (%) and (**D**) aqueous-fraction yield (mg g_biomass_^−1^ DW) during solid-liquid-liquid extraction of disrupted wet *Phaeodactylum tricornutum biomass* using ethyl acetate–n-butanol–water (3:2:5, *v*/*v*/*v*) at 25 °C (disintegration degree > 95%).

The fitted response-surface models adequately described the main responses, including lipophilic fraction yield, aqueous fraction yield, fucoxanthin recovery and EPA recovery (Figure 3A–D), while the emulsion and pellet responses are shown in Supplementary Material (Figure S1 and S2). Model quality was assessed using the coefficient of determination (R^2^) as a measure of goodness-of-fit and the cross-validated predictability coefficient (Q^2^) as a measure of predictive ability. Across the main responses, R^2^ values ranged from 0.782 to 0.934 and Q^2^ values from 0.647 to 0.892, meeting established Design of Experiments (DoE) quality criteria and indicating satisfactory model performance. In addition, no problematic multicollinearity was detected, as indicated by the low condition number [44]. Detailed statistical parameters, model equations and analysis-of-variance results are provided in Supplementary Material (Table S1 and S2).

The lipophilic fraction yield provides an overview of the total material transferred into the clear organic-rich phase before considering individual target compounds (Figure 3A). The response surface indicates that the overall mass recovered in this phase was mainly controlled by DW content and showed a broad maximum at relatively low biomass loading and intermediate extraction time. The highest lipophilic fraction yield (321 mg g_biomass_^−1^) was predicted around 2.9 h and 1.0% DW. Longer extraction therefore did not necessarily increase the total recoverable organic-phase mass. Instead, prolonged extraction, particularly at higher DW contents, may have promoted the accumulation of amphiphilic biomass-derived compounds at the liquid–liquid interface rather than their transfer into the clear lipophilic phase. This interpretation is supported by the emulsion response shown in Supplementary Material (Figure S1) and is consistent with the interfacial activity of polar membrane lipids and phospholipids in aqueous–organic systems [45,46].

Within the lipophilic phase, fucoxanthin and EPA recoveries were both governed by extraction time and DW content, but the two target compounds showed different sensitivities to biomass loading (Figure 3B,C). Here, recovery refers to the proportion of the initial mass of the target compound recovered in the lipophilic phase. The disrupted biomass initially contained 15 mg g^−1^ DW fucoxanthin and 38 mg g^−1^ DW EPA on average. For both responses, longer extraction improved recovery mainly at low and intermediate DW contents, whereas high DW contents limited recovery even when extraction time was increased. The stronger decline in recovery at high DW contents indicates that transfer into the clear lipophilic phase became increasingly hindered, likely because reduced solvent availability promoted accumulation of biomass-derived material at the aqueous–organic interface. The statistical parameters supporting these effects are provided in Supplementary Material (Tables S1 and S2). Fucoxanthin reached higher recovery and tolerated higher biomass loading than EPA. The model-predicted fucoxanthin optimum was located near 5.0 h and 3.70% DW, corresponding to approximately 99% recovery and a solvent-to-biomass ratio of about 28:1 (*v*/*w*). Fucoxanthin recovery remained close to the optimum, above approximately 90–95%, around 5% DW, corresponding to an approximately 20:1 (*v*/*w*) solvent-to-biomass ratio, but decreased markedly above about 7–8% DW, where the solvent-to-biomass ratio dropped to approximately 14:1–12.5:1 (*v*/*w*). In contrast, EPA recovery reached its maximum at lower biomass loading, near 4.9 h and 1.7% DW, corresponding to 84–85% recovery and a solvent-to-biomass ratio of 59:1 (*v*/*w*). Accordingly, the model-predicted optimal recoveries correspond to approximately 14.9 mg fucoxanthin g^−1^ initial biomass DW and 31.9–32.3 mg EPA g^−1^ initial biomass DW recovered in the lipophilic phase.

Overall, the ethyl acetate/n-butanol/water SLLE system is therefore particularly effective for producing a fucoxanthin-rich lipophilic fraction with substantial EPA co-recovery in a single extraction step, while the EPA response highlights the importance of controlling DW content, solvent availability and interfacial phase behaviour when targeting membrane-bound lipids. This difference indicates that EPA-containing lipids required a higher solvent-to-biomass ratio (*v*/*w*) for efficient transfer into the clear lipophilic phase. Although these precautions support the reliable comparison of extraction conditions within the applied analytical workflow, oxidation products were not specifically quantified. Therefore, the reported EPA and fucoxanthin recoveries are best interpreted as apparent recoveries of the quantified intact target compounds. This also provides a useful direction for future process refinement, where additional control of oxygen exposure, light, and temperature, together with monitoring of lipid and pigment oxidation markers, could increase EPA and fucoxanthin recoveries.

The different behaviours of fucoxanthin and EPA observed in the present SLLE system are consistent with the solvent-dependent extraction trends reported by Delbrut et al. for *P. tricornutum* [47]. It should be noted that this comparison is not intended as a direct one-to-one comparison between pure ethyl acetate extraction and the present biphasic SLLE system, in which ethyl acetate was used as one component of a designed ethyl acetate/n-butanol/water solvent system. In their solvent screening, pure ethyl acetate showed limited extraction performance, reaching only about 42% fucoxanthin recovery relative to the methanol reference after 1 h. For EPA, ethyl acetate was likewise inefficient during the short extraction screening, reaching only approximately 25–30% recovery after 90 min. In line with this low initial performance, the research conducted by Delbrut et al. did not further investigate prolonged ethyl acetate extraction conditions approaching high recovery [47]. Instead, extended recovery experiments focused mainly on alcohol-based systems: 96% ethanol reached 95% fucoxanthin recovery after 24 h at a 20:1 (*v*/*w*) solvent-to-biomass ratio and 89% EPA recovery after 8 h at the same ratio.

Against this background, the present results show that an ethyl acetate-containing solvent system can become highly effective when ethyl acetate is combined with n-butanol and water in a designed biphasic SLLE system, rather than being applied as a standalone solvent. The present system reached approximately 99% fucoxanthin recovery and 84–85% EPA recovery after 5.0 h in a single solvent–biomass contact, while simultaneously generating a separated lipophilic product phase. Thus, the process gain is not simply high extraction efficiency, but the transformation of a solvent with limited standalone performance into an effective integrated fractionation environment through solvent-system design.

The improved performance is likely linked mainly to the complementary roles of the three solvent components and their combined influence on solvent polarity, amphiphilic interactions, and partition behaviour. Ethyl acetate promotes partitioning of lipophilic compounds into the organic phase, while n-butanol adds amphiphilic character and hydrogen-bond donor/acceptor capacity. Water adjusts the overall polarity of the system and supports biphasic separation, thereby influencing the distribution of compounds between the lipophilic phase, aqueous phase, and interface. This polarity-balanced environment is particularly relevant for fucoxanthin, which is an oxygenated xanthophyll containing epoxy, carbonyl and hydroxyl groups. Sun et al. similarly reported that controlled water content improved ethanol-based fucoxanthin extraction from *P. tricornutum* [48]. At the same time, the previously achieved cellular disintegration above 95% should still be regarded as an important enabling pretreatment, because it reduced physical mass-transfer barriers before extraction; however, the improved SLLE performance is mainly attributed to the polarity-balanced mixed-solvent environment rather than to additional cell disruption during extraction. Given this high degree of prior disruption, the favourable fucoxanthin recovery likely reflects efficient solvent-mediated release of fucoxanthin from fucoxanthin–chlorophyll a/c-binding protein complexes associated with thylakoid membranes, followed by partitioning into the lipophilic phase [49,50]. The relevance of butanol-type solvents is further supported by the work of König-Mattern et al., where it was shown that partially water-miscible butanol systems can be effective for wet *P. tricornutum* fractionation [25].

For EPA, the outcome also reveals an important process limitation. Although substantial EPA co-recovery was achieved, the EPA optimum shifted to lower DW content and a higher solvent-to-biomass ratio (*v*/*w*) than fucoxanthin. This suggests that the biphasic concept is highly effective for fucoxanthin partitioning, but less ideal for unrestricted transfer of EPA-containing glycerolipids. Unlike fucoxanthin, EPA is mainly recovered as part of native lipid classes rather than as a free compound. In *P. tricornutum*, EPA is incorporated into glycerolipids, including amphiphilic glycolipids such as monogalactosyldiacylglycerol, digalactosyldiacylglycerol and sulfoquinovosyldiacyl-glycerol. These EPA-containing membrane lipids can adsorb at the aqueous–organic interface in the ethyl acetate/n-butanol/water system, establishing an emulsion layer and limiting transfer into the clear lipophilic phase [51]. This explains why EPA required lower DW content and higher solvent availability than fucoxanthin, and why the total lipophilic fraction did not simply increase with extraction time even when compound-specific recoveries improved.

The aqueous fraction showed a predominantly DW-content-dependent response, whereas extraction time exerted only a minor influence on the recovered aqueous mass (Figure 3D). Detailed statistical parameters supporting these trends are provided in Supplementary Material (Tables S1 and S2). The aqueous fraction yield decreased from approximately 0.33–0.35 g g_biomass_^−1^ DW at low DW contents to 0.21–0.24 g g_biomass_^−1^ DW at the highest DW contents. This decline indicates that recovery of water-soluble material was mainly constrained by the increasing biomass concentration in the SLLE system rather than by extraction duration. At elevated DW contents, the reduced solvent availability per unit biomass, increased slurry viscosity and less efficient phase disengagement likely hindered transfer of soluble compounds into the clear aqueous phase, consistent with observations reported for concentrated microalgal slurries and wet extraction systems [24,52,53].

The interfacial fraction showed the opposite trend, increasing most strongly when prolonged extraction was combined with high DW content (Supplementary Material, Figure S1). This indicates that extended contact under concentrated conditions promoted the accumulation of biomass-derived material at the liquid–liquid interface rather than its recovery in the clear product phases. Such interfacial enrichment is expected in disrupted microalgal systems, where proteins, polar lipids and fine cell debris can adsorb at solvent–water interfaces and stabilise emulsion layers [45,53,54,55]. In the present system, this behaviour is process-relevant because interfacial accumulation can reduce the recoverable mass in both the lipophilic and aqueous fractions and helps explain why longer extraction did not proportionally increase the total lipophilic fraction yield.

The residual pellet represented a different limitation. Its yield was highest at high DW content and short extraction time (Supplementary Material, Figure S2), indicating incomplete release of biomass components when the solvent-to-biomass ratio (*v*/*w*) and contact time were insufficient. Under these conditions, part of the disrupted biomass remained associated with the insoluble residue rather than being transferred into the aqueous, lipophilic or interfacial fractions. Similar behaviour has been described for concentrated or insufficiently extracted microalgal biomass, where limited solvent penetration and incomplete solubilization reduce overall recovery [45,52,56].

Overall, biomass content was the dominant process variable, while extraction time acted as a secondary parameter. Longer extraction time improved fucoxanthin and EPA recovery but did not proportionally increase the total lipophilic mass. Increasing biomass content, by contrast, negatively affected all key responses by imposing transport and phase-separation constraints. From a process perspective, these findings indicate that increasing biomass loading at constant solvent ratios does not result in a proportional improvement in extraction performance, suggesting concentration-dependent limitations in phase capacity and solute partitioning. Consequently, scale-up should be based on staged or multi-step extraction schemes with intermediate phase separation, rather than on a single high-loading extraction step, to maintain favourable partitioning conditions, reduce solvent-phase saturation, and enhance recovery across the target and co-product streams.

#### 2.1.2. Selection of an Operating Window

As *P. tricornutum* is considered a multiproduct biorefinery feedstock, process optimisation must consider overall product distribution rather than single-compound recovery alone. Fucoxanthin represents the primary value-driving compound, while EPA and hydrophilic constituents form important co-products [57]. Optimisation was therefore directed towards identifying operating conditions that maintain high fucoxanthin recovery while preserving relevant co-product fractions and improving batch productivity.

The fitted fucoxanthin response surface methodology (RSM) model predicted the maximum-recovery condition, 99%, at approximately 5.0 h extraction time and 3.70% biomass content (Figure 3B). However, this condition should be interpreted as the upper recovery benchmark of the model rather than as the optimal operating condition. As indicated by the shallow response surface in Figure 3B, fucoxanthin recovery at approximately 3.70% biomass content had already reached about 90% after 1 h. Extending the extraction from 1.0 to 5.0 h increased the predicted recovery to approximately 99%, corresponding to an average recovery increase of approximately 2.25 percentage points h^−1^ between 1.0 and 5.0 h extraction time. In batch extraction, this marginal gain must be weighed against the longer cycle duration, since extended extraction times reduce the number of batches that can be processed within a fixed operating period. Thus, under the single-stage batch productivity criterion used here, the additional recovery achieved near the maximum-recovery benchmark does not compensate for the loss in batch throughput. The optimisation was therefore shifted from exhaustive single-batch recovery towards conditions that combine high product recovery with shorter extraction time, suitable biomass loading, and preservation of relevant co-product fractions.

Thus, the operating window was selected using a two-stage constrained optimisation procedure. First, a lexicographic feasibility screen was applied to retain only model-predicted conditions fulfilling minimum acceptable process criteria. This screen acted as a hard filter before desirability ranking. Its purpose was to avoid selecting conditions that appeared highly productive mainly because of short extraction time or high biomass loading but did not provide sufficient product recovery or acceptable fraction distribution. In other words, high calculated productivity alone was not considered sufficient if fucoxanthin recovery, EPA recovery, co-product fraction yields, or residual-solid control fell below the predefined process requirements. The feasibility screen therefore ensured that only conditions providing acceptable multiproduct performance within a single-stage batch extraction were considered for the subsequent ranking.

The feasibility thresholds were selected according to product relevance and the experimentally attainable response ranges observed within the DoE, rather than as universal literature limits. Fucoxanthin recovery was constrained to ≥90% because fucoxanthin is the main value-driving product and this recovery level was attainable within the investigated design space. EPA recovery was constrained to ≥70% because EPA showed a lower attainable recovery range; recoveries close to 90% were not reached in the DoE, so applying the same threshold as for fucoxanthin would have removed the feasible operating region. Minimum lipophilic and hydrophilic fraction yields of ≥0.165 and ≥0.295 g g_biomass_^−1^ DW, respectively, were imposed to preserve the multiproduct character of the process, while the pellet fraction was limited to ≤0.43 g g_biomass_^−1^ DW to avoid conditions with excessive residual solids, thus decreasing overall biomass valorisation.

Only conditions fulfilling all feasibility criteria were then ranked using a weighted desirability function based on throughput-adjusted productivity terms. This approach follows the Derringer–Suich multi-response optimisation framework, in which individual responses are transformed into desirability values and combined into an overall score [58]. Because multi-response optimisation involves trade-offs between partly competing objectives, the weights were used as a semi-quantitative representation of product hierarchy and process-operability priorities rather than as fixed economic coefficients [59]. Fucoxanthin and EPA productivities received the highest weights, 0.35 and 0.30, respectively, reflecting their role as the principal target products of the *P. tricornutum* biorefinery. The lipophilic and hydrophilic fractions were assigned lower positive weights, 0.15 and 0.10, respectively, to preserve broader co-product recovery without allowing less defined bulk fractions to dominate the ranking. Pellet and emulsion/interlayer formation were included as penalised responses with weights of 0.06 and 0.04, respectively, because they indicate reduced biomass utilisation and impaired phase behaviour. Extraction time and biomass loading were not assigned separate desirability weights because both were already incorporated into the productivity expressions, avoiding double counting of the same engineering effects.

This process-oriented optimisation did not identify a single isolated optimum, but rather a narrow operating window. As summarised in Supplementary Material (Table S3), the ten highest-ranked candidates were located between approximately 1.25–1.45 h extraction time and 4.00–4.65% biomass content, with only a 2.2% relative desirability difference between the first- and tenth-ranked solutions. This indicates that the selected region was relatively robust and not the result of a single numerical artefact. The highest-ranked condition, 1.3 h extraction time and 4.25% biomass content, was therefore selected as the nominal operating condition (Supplementary Material, Table S3, Rank 1).

At this condition, the models predicted 91.3% fucoxanthin recovery, 70.0% EPA recovery, 0.168 g g_biomass_^−1^ DW lipophilic fraction, 0.302 g g_biomass_^−1^ DW hydrophilic fraction, 0.107 g g_biomass_^−1^ DW emulsion/interlayer, and 0.423 g g_biomass_^−1^ DW pellet fraction. The selected condition therefore fulfilled all predefined feasibility criteria. Notably, the pellet fraction remained just below the imposed upper limit, indicating that this constraint was relevant in preventing the selection of conditions that could have increased apparent productivity but reduced processability.

Compared with the maximum-recovery benchmark at 5.0 h and 3.70% biomass content (Figure 3A–D), the selected operating condition reduced extraction time by 74% and increased biomass content from 3.70% to 4.25%. The associated decrease in single-batch recovery was moderate: fucoxanthin recovery decreased by approximately 7.7 percentage points, from approximately 99.0% to 91.3%, while EPA recovery decreased by 11.8 percentage points. The hydrophilic fraction yield decreased by 5.6%, whereas the lipophilic fraction increased by 7.7% and the emulsion/interlayer fraction decreased by 37%. Although the pellet fraction increased by 21%, it remained within the predefined feasibility limit and was therefore considered an acceptable trade-off in view of the substantial gain in batch productivity.

When process performance was evaluated per unit extraction time, the advantage of the selected operating condition became evident. Predicted fucoxanthin productivity increased from 0.110 to 0.448 mg g_suspension_^−1^ h^−1^, corresponding to a 4.1-fold enhancement. Similarly, EPA productivity increased from 0.222 to 0.870 mg g_suspension_^−1^ h^−1^, representing a 3.9-fold gain. Productivity of the lipophilic fraction increased from 1.15 to 5.49 mg g_suspension_^−1^ h^−1^, while hydrophilic fraction productivity increased from 2.35 to 9.87 mg g_suspension_^−1^ h^−1^. These increases reflect the combined effect of shorter extraction time and higher biomass loading. Therefore, despite the lower recovery per individual batch, the amount of recovered product per extraction hour is markedly higher.

The selected operating condition should therefore be interpreted in the context of single-stage batch extraction. For scale-up, cascade extraction could represent a promising strategy to further increase cumulative recovery and overall productivity, particularly if valuable compounds remain in residual pellet or emulsion/interlayer fractions after the first extraction stage. Such an approach would require an expanded optimisation framework that includes the additional recovery obtained in each extraction stage, repeated phase separation or centrifugation, solvent recycling or replacement, re-extraction time, and the resulting total process time. However, this strategy is worth further investigation as a potential route to improve overall biomass utilisation.

Thus, the 5.0 h and 3.70% biomass condition defines the upper recovery benchmark of the model, whereas 1.3 h and 4.25% biomass content represent the more relevant optimal operating condition for single-stage batch processing. This condition maintains high fucoxanthin recovery, preserves EPA and co-product fraction recovery above the predefined feasibility limits, and substantially improves batch throughput, biomass utilisation, and solvent-use efficiency.

#### 2.1.3. Process Performance Under Optimised Conditions

The selected setpoint, 1.3 h extraction time and 4.25% biomass content, was experimentally evaluated to verify model predictions and assess the composition of the resulting fractions. Three descriptors were used: phase yield, expressed as the volumetric distribution of the separated phases (*v*/*v*); biomass-normalised recovery, expressed as mg recovered material per g initial biomass DW; and fraction-specific contents, expressed as mg component per g dry matter of the respective fraction. These data are summarised in Table 1. Direct comparison with previous studies should be interpreted with caution, because most published work on *P. tricornutum* reports either whole-biomass composition or stepwise recovery of selected target compounds rather than dry-matter composition of each recovered fraction [30,60,61].

The RSM model predicted recoveries of 168 mg g_biomass_^−1^ DW lipophilic extract, 107 mg g_biomass_^−1^ DW interfacial fraction, 302 mg g_biomass_^−1^ DW aqueous extract, and 423 mg g_biomass_^−1^ DW insoluble residue. Experimentally, the corresponding values were 165 ± 11, 113 ± 7, 303 ± 18, and 418 ± 25 mg g_biomass_^−1^ DW, respectively, resulting in a closed mass balance of 1,000 mg g_biomass_^−1^ DW. Deviations from model predictions were small, namely −1.7%, +5.8%, +0.5%, and −1.1%, confirming good predictive performance. The corresponding phase yields were 0.45 ± 0.02 for the lipophilic phase, 0.05 ± 0.01 for the interfacial layer, 0.39 ± 0.02 for the aqueous phase, and 0.11 ± 0.02 for the insoluble residue, indicating that most of the system volume was distributed between the two bulk liquid phases.

Biochemical analysis revealed clear compositional differences between the fractions (Table 1). The lipophilic fraction was dominated by glycerolipids. On the dry matter basis of the lipophilic extract, glycerolipids accounted for 719 ± 66 mg g_fraction_^−1^, followed by fucoxanthin (85.3 ± 7.8 mg g_fraction_^−1^) and chlorophyll a (82.3 ± 7.6 mg g_fraction_^−1^). Chlorophyll c, diadinoxanthin, β-carotene, lutein, zeaxanthin, lipophilic polyphenols, and unidentified lipophilic compounds accounted for the remaining extract mass. On a biomass basis, glycerolipid recovery in this fraction alone was 119 ± 8 mg g_biomass_^−1^ DW, corresponding to 11.9% of the initial biomass DW, which is of the same order as reported for *P. tricornutum* extracts [10]. Because the lipophilic extract represented 165 mg per g biomass DW, quantitative transfer of a compound into this fraction would correspond to a theoretical concentration factor of approximately 6.1.

The accumulation of more than 70% of total EPA in this phase therefore corresponds to a minimum enrichment of approximately 4.2-fold. Fucoxanthin showed the same preferential partitioning, with a biomass-normalised recovery of 14.1 ± 0.89 mg g_biomass_^−1^ DW. In addition, the fucoxanthin content of the lipophilic extract, 85.3 ± 7.8 mg g_fraction_^−1^ DM, was approximately 5.3-fold higher than the 16.1 mg g^−1^ dried extract reported by a previous study for a crude *P. tricornutum* extract entering high-performance counter current chromatography (HPCCC) purification [35]. Thus, the SLLE already provides substantial pre-enrichment prior to chromatographic purification.

The aqueous fraction accounted for 303 ± 108 mg g_biomass_^−1^ DW and was dominated by water-soluble carbohydrates (544 ± 47 mg g_fraction_^−1^ DW) followed by soluble proteins and peptides (165 ± 14 mg g_fraction_^−1^ DW), unidentified hydrophilic metabolites (160 ± 14 mg g_fraction_^−1^ DW), ash (132 ± 11 mg g_fraction_^−1^ DW) and hydrophilic polyphenols (3.3 ± 0.4 mg g_fraction_^−1^ GAE). This composition reflects the expected enrichment of hydrophilic compounds after cell disruption and demonstrates that the aqueous phase constitutes a relevant co-product stream rather than merely a residual fraction. In particular, the presence of carbohydrates, proteins, and minerals indicates that this stream may provide functional value beyond mass recovery, for example, as a source of water-soluble bioactive compounds, techno-functional ingredients such as emulsifiers, or fermentation substrates [21,62,63]. This functional potential is supported by previous work on *P. tricornutum*, where carbohydrate- and protein-rich aqueous extracts were shown to exhibit gel-forming properties [10].

Although the interfacial fraction represented only a small phase volume (Figure 2), it accounted for 113 ± 7 mg g_biomass_^−1^ DW and showed a mixed composition (Table 1). On a DW basis, it contained interface-associated proteins (353 ± 31 mg g_fraction_^−1^), unidentified interfacial material (302 ± 26 mg g_fraction_^−1^), glycerolipids (266 ± 23 mg g_fraction_^−1^) and non-glycerolipid-like lipophilic compounds (79.5 ± 6.9 mg g_fraction_^−1^). This protein content is close to the 37.2 DW% reported previously for water-soluble *P. tricornutum* protein extracts with interfacial activity, although the present fraction is compositionally more complex because it additionally contains lipids and unidentified material [64]. The observed composition supports its interpretation as a membrane-protein-rich amphiphilic fraction rather than as simple separation waste. This is consistent with the behaviour described before, where it was shown that, during wet microalgal extraction, protein-rich serum can form a strong interfacial film and cell debris can adsorb to droplet surfaces; thus, the co-presence of lipids and debris modifies emulsion stability at the oil–water interface [45]. The retention of residual EPA and fucoxanthin in this layer is also mechanistically plausible, because EPA is associated with membrane lipids and fucoxanthin occurs in fucoxanthin–chlorophyll protein complexes of the photosynthetic apparatus [65,66]. Accordingly, despite the substantial proportion of material not assigned by the targeted compositional analyses, the interfacial fraction should not be regarded merely as separation waste, but rather as a complex amphiphile-rich co-product stream with potential for further valorisation. This interpretation is supported by reports showing that algae biomass, extracts, and extract-enriched ingredients are already used or evaluated in cosmetic and formulation-related contexts [67,68,69], and by safety assessments noting that algae-derived cosmetic ingredients may contain hundreds of constituents and are therefore assessed as whole, complex mixtures rather than as fully defined single compounds [70]. Nevertheless, further chemical and functional characterisation, particularly of the unidentified material and its emulsifying or stabilising properties, is required before specific application-related conclusions can be drawn. After such characterisation, the fraction could be further evaluated either as an amphiphilic emulsifying or stabilising system for cosmetic, food, or feed formulations, or as a feedstock for further recovery and valorisation of bioactive compounds, for example through enzymatic hydrolysis of proteinaceous components to obtain peptide-rich hydrolysates.

The insoluble residue (Figure 2) accounted for 418 ± 25 mg g_biomass_^−1^ DW. On a DW basis, it was dominated by polysaccharides (359 ± 31 mg g_fraction_^−1^), followed by proteins, (263 ± 22 mg g_fraction_^−1^), unidentified insoluble material (187 ± 16 mg g_fraction_^−1^), ash (132 ± 11 mg g_fraction_^−1^) and residual lipophilic compounds (59.8 ± 5.1 mg g_fraction_^−1^). The residue was therefore depleted in readily extractable lipophilic material but enriched in carbohydrate-type material. The persistence of a polysaccharide-rich residue despite the carbohydrate-rich aqueous phase likely reflects the carbohydrate architecture of *P. tricornutum*. Caballero distinguished soluble and insoluble carbohydrate pools in this diatom, while Castro-Ferreira reported cell-wall polysaccharides, including sulphated glucuronomannan [10,71]. The residue can therefore be interpreted as a structurally biased secondary biomass fraction rather than a process-related loss.

### 2.2. Purification via CPC

CPC was applied to the lipophilic extract as a staged fractionation platform rather than as a single-target fucoxanthin purification method. This distinction is important because most reported LLC studies on algal matrices, including micro- and macroalgae, have focused mainly on the isolation of individual carotenoids [37,38,40,72], particularly fucoxanthin [34,36,73], due to its high commercial value (40,000–80,000 US$ kg^−1^ [17]) and reported bioactivities.

However, in the present work, the lipophilic extract was not regarded solely as a source of fucoxanthin, but as a source of several potential product classes, including EPA-enriched glycerolipids, carotenoids, chlorophylls, polyphenolic compounds, and neutral lipids. This broader process objective required a chromatographic strategy capable of both fractionating the crude extract into compositionally distinct regions and improving the purity of selected target compounds. The crude lipophilic extract contained compounds spanning a wide polarity range, from relatively polar phenolic compounds and amphiphilic glycerolipids to pigments and neutral lipids. Therefore, a single CPC run was unlikely to provide both broad extract fractionation and complete fucoxanthin purification.

For this reason, the process was designed as a two-stage CPC sequence. The first CPC run was applied to the crude lipophilic extract to divide it into major product regions, while the second CPC run was applied only to the fucoxanthin-containing intermediate fraction to achieve improved selectivity and purification. Prior to CPC purification, several biphasic solvent systems were screened in preliminary liquid–liquid partition experiments. The final solvent systems were selected and adjusted based on the partition coefficient ($K_{D}$) values of the target compounds, since $K_{D}$ governs analyte distribution between the stationary and mobile phases and therefore directly influences retention, selectivity, resolution, and purification efficiency.

Based on this screening, two quaternary alkane/ethyl acetate/alcohol/water solvent systems were selected to investigate different partitioning and selectivity windows for the staged CPC fractionation of the SLLE-derived lipophilic extract. Such solvent systems are widely used in CCC and CPC because their polarity and selectivity can be systematically adjusted by varying the alkane/ethyl acetate and alcohol/water ratios [74,75,76]. The first CPC run employed n-hexane/ethyl acetate/methanol/water (HEMWat) (8:2:8:2, *v*/*v*/*v*/*v*), corresponding to a classical HEMWat-type system. This alkane-rich composition was selected to favour the partitioning of relatively lipophilic constituents while retaining ethyl acetate and water as polarity-modulating components. The 8:2 ratio in both the n-hexane/ethyl acetate and methanol/water pairs was used to maintain the systematic HEMWat composition principle while screening a comparatively low-polarity solvent system. In contrast, the second CPC run employed n-heptane/ethyl acetate/ethanol/water (5:5:5.4:4.6, *v*/*v*/*v*/*v*) as a more balanced, modified Arizona-type system [76]. The 5:5 n-heptane/ethyl acetate ratio increased the contribution of the moderately polar ester component in the organic solvent-rich phase, whereas the near-equivalent ethanol/water ratio was used to adjust the polarity and hydrogen-bonding character of the aqueous phase while maintaining biphasic behaviour. This second solvent system was therefore included to shift the partitioning of more polar or amphiphilic constituents and to provide an alternative selectivity compared with the alkane-rich HEMWat system. Although conventional alkane-based solvent systems were used in the present study, the preliminary liquid–liquid partitioning/solvent-system screening and subsequent CPC fractionation stages represent clear opportunities for future replacement of n-hexane or n-heptane with greener solvent systems, including d-limonene-based alkane substitutes and more recently proposed 2-methyltetrahydrofuran-, ethyl isobutyrate-, or hexamethyldisiloxane-containing biphasic systems [77,78,79,80].

Applied solvent systems in this study are chemically related to previously reported CPC/HPCCC systems for fucoxanthin purification. The first system is comparable to the n-hexane–ethyl acetate–ethanol–water system reported by Kim et al., although methanol was used here instead of ethanol [73]. The second system is more directly comparable to the n-heptane–ethyl acetate–ethanol–water system used by Bárcenas-Pérez since both are based on the same solvent components [35]. In contrast, the cyclohexane–alcohol–water systems reported by De Oliveira-Júnior and Pajot are less directly comparable because they do not contain ethyl acetate [34,36].

These previous studies therefore provide useful reference points for the present work, although direct benchmarking is limited by differences in process objective and purity definition. Kim et al., for example, first generated a fucoxanthin-enriched fraction from *Eisenia bicyclis* and then required two CPC steps to increase purity from approximately 81% to >98% [73]. De Oliveira-Júnior obtained fucoxanthin with 65.7% purity after CPC separation of *Tisochrysis lutea* extracts and reached >99% purity only after coupling CPC with flash chromatography [36]. Bárcenas-Pérez et al. achieved 97% purity and 98% recovery using HPCCC with multiple sequential injections, while Pajot reported 100% high-performance liquid chromatography (HPLC)-based pigment purity and 92% recovery after CPC solvent-system optimisation [34,35]. However, in the latter case, purity was defined based on pigment analysis by HPLC and did not account for co-extracted lipids, which limits comparison with whole-fraction compositional purity. Against this background, the present CPC workflow was assessed not by final fucoxanthin purity, but also by its capacity to generate multiple compositionally distinct lipophilic fractions.

#### 2.2.1. First CPC Run

The first CPC separation was designed to process the complete crude lipophilic extract and to divide it into chemically distinct product regions. The solvent system n-hexane–ethyl acetate–methanol–water (8:2:8:2, *v*/*v*/*v*/*v*) was selected because it provided stable hydrodynamic behaviour and broad polarity-based fractionation. The run was performed with 200 mg crude extract on a 247 mL rotor, corresponding to a column loading of approximately 0.81 mg mL^−1^. This loading was higher than that reported for one-step elution–extrusion counter-current chromatographic purification of fucoxanthin from brown algae by Chen et al. (60 mg on 310 mL; approximately 0.19 mg mL^−1^ [81]), but lower than the HPCCC loading applied by Bárcenas-Pérez (240 mg on 134 mL; approximately 1.8 mg mL^−1^ [35]). Thus, the applied loading was within the preparative range reported for related LLC separations.

The system showed excellent phase retention during descending-mode operation, with a stationary phase retention ($S_{f}$) of 0.83 ± 0.02 at a flow rate of 5 mL min^−1^. This confirmed that the separation was not limited by poor hydrodynamic stability. The distribution coefficients further explained the elution sequence: polyphenolics showed a low $K_{D}$ of approximately 0.07, EPA-enriched glycerolipids showed an intermediate $K_{D}$ of approximately 0.50, whereas chlorophyll *c*, fucoxanthin, and diadinoxanthin occupied a narrow pigment region with $K_{D}$ values of 1.05, 1.15, and 1.30, respectively. The close spacing between these pigment $K_{D}$ values indicated limited selectivity within this central region, even though each individual $K_{D}$ value was within a suitable CPC operating range.

**Figure 4:**
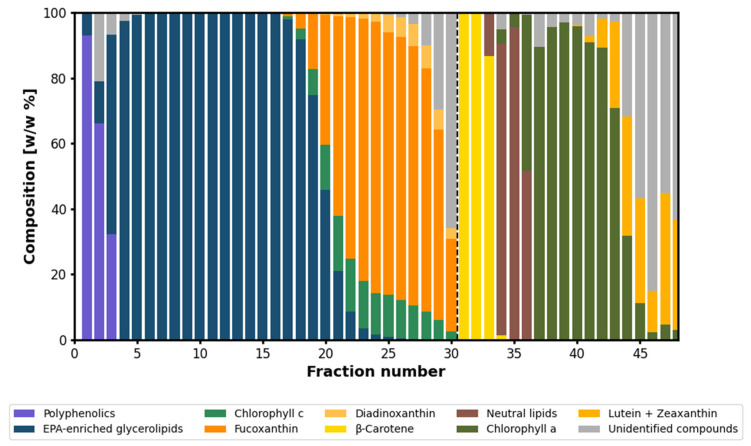
Composition of individual fractions obtained during the first centrifugal partition chromatography (CPC) run, expressed as relative abundance (*w*/*w*%, dry weight (DW) based). Values shown in the stacked bars represent mean values calculated from technical replicate CPC runs performed under the same operating conditions (n = 3). Fractions are shown as a function of fraction number, highlighting the sequential elution of polyphenolics, eicosapentaenoic acid (EPA)-enriched glycerolipids, fucoxanthin, chlorophyll c, diadinoxanthin, β-carotene, neutral lipids, chlorophyll a, lutein and zeaxanthin, and unidentified compounds. The dotted line at fraction number 30 denotes the transition from descending to ascending mode. An n-hexane/ethyl acetate/methanol/water (8:2:8:2, *v*/*v*/*v*/*v*) biphasic solvent system was employed for primary fractionation of the crude lipophilic extract. The upper phase was used as the stationary phase in descending mode. The rotor was filled at 20 mL min^−1^ and 55× g followed by acceleration to 717× *g* for 10 min to establish hydrodynamic equilibrium. The mobile phase (lower phase) was then introduced at 5 mL min^−1^. A total of 200 mg of crude lipophilic extract was loaded.

These partitioning differences are reflected in Figure 4. Fractions 1–3 yielded a polyphenolic-rich fraction with a purity of 68.7 ± 4.0%. This early elution was consistent with the low $K_{D}$ of the phenolic compounds and their preferential distribution into the mobile phase under the selected operating mode. Fractions 4–16 (Figure 4) then yielded an EPA-enriched glycerolipid fraction with a purity of 99.9 ± 0.5%. This was one of the most important outcomes of the first CPC run, because EPA-containing glycerolipids are often treated as co-extracted matrix components in fucoxanthin purification workflows. Here, they were recovered as a highly selective product fraction before the main pigment region. The central chromatographic region, fractions 17–29 (Figure 4), contained fucoxanthin. Within this region, fractions 23–26 showed the highest enrichment, with a maximum estimated fucoxanthin purity of 84.0 ± 1.5% in fraction 24. However, complete isolation of fucoxanthin was not achieved in this first run because chlorophyll c eluted slightly earlier but overlapped strongly with fucoxanthin, while diadinoxanthin became increasingly prominent in the later part of the same region. This behaviour is directly explained by the similar $K_{D}$ values of the three pigments. Under preparative loading conditions, such closely spaced $K_{D}$ values are sufficient to concentrate fucoxanthin into a defined region, but not sufficient to fully resolve it from pigments with highly similar polarity. For this reason, fractions 17–29 were pooled as a fucoxanthin-containing intermediate rather than being treated as the final purified product. The pooled fraction reached a fucoxanthin purity of 45.4 ± 3.5%. Although this value is lower than the maximum purity observed in individual fractions, pooling preserved recovery and generated enough material for the second CPC step. Therefore, this approach was capture-oriented. It removed early-eluting glycerolipids and late-eluting hydrophobic compounds while concentrating fucoxanthin into a narrower intermediate fraction.

After the fucoxanthin-containing region had eluted, the CPC operation was switched from descending to ascending mode (Figure 4, dotted line). This phase-role inversion enabled retained analytes in the stationary phase to be mobilised during the subsequent separation stage without transfer to a separate chromatographic system. As a result, more hydrophobic components were recovered sequentially. Fractions 31–32 (Figure 4) yielded a β-carotene-enriched fraction with a purity of 73.9 ± 3.0%, followed by a neutral lipid fraction dominated by triacylglycerols (TAGs), diacylglycerols (DAGs), and monoacylglycerols (MAGs) in fractions 34–36 (Figure 4), with a purity of 97.4 ± 1.5%. Fractions 37–42 yielded a chlorophyll a-rich fraction with an average purity of 95.1 ± 0.7%, while fractions 43–48 contained mainly lutein/zeaxanthin with substantial amount of unidentified compounds (Figure 4).

#### 2.2.2. Second CPC Run

The second CPC run was applied specifically to the fucoxanthin-containing intermediate obtained from the first separation. The objective of the second separation was therefore to improve selectivity within the pigment region, resulting in improved separation. For this purpose, the solvent system n-heptane–ethyl acetate–ethanol–water (5:5:5.4:4.6, *v*/*v*/*v*/*v*) was selected. This system changed the partitioning of the remaining components favourably. The $K_{D}$ values were 0.22 for residual glycerolipids, 0.78 for chlorophyll c, 1.33 for fucoxanthin, and 2.00 for diadinoxanthin. Compared with the first solvent system, the selectivity between the key pigment compounds was substantially improved and provided the chromatographic basis for resolving the previously overlapping pigment cluster.

**Figure 5:**
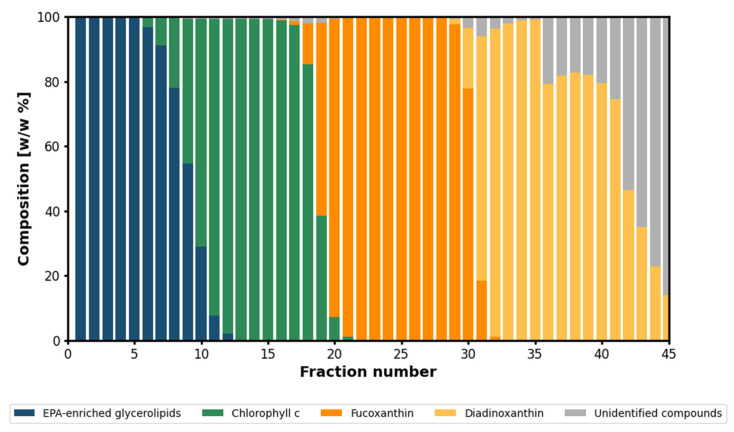
Composition of individual fractions obtained during the second centrifugal partition chromatography (CPC) run, expressed as relative abundance (*w*/*w*%, dry weight (DW) based). Values shown in the stacked bars represent mean values calculated from technical replicate CPC runs performed under the same operating conditions (n = 3). Fractions are shown as a function of fraction number, highlighting the sequential elution of eicosapentaenoic acid (EPA)-enriched glycerolipids, chlorophyll c, fucoxanthin, diadinoxanthin, and unidentified compounds. The fucoxanthin-enriched fraction (200 mg) obtained from the first CPC run was reconstituted in the lower phase of an n-heptane/ethyl acetate/ethanol/water (5:5:5.4:4.6, *v*/*v*/*v*/*v*) biphasic solvent system and subjected to a second CPC separation. The rotor was filled at 20 mL min^−1^ and 55× *g* in descending mode, followed by acceleration to 717× *g* to establish hydrodynamic equilibrium. The mobile phase (lower phase) was then introduced at 4 mL min^−1^. No phase inversion was applied during this run.

Distinct product regions were obtained during the separation process (Figure 5). Early fractions contained residual EPA-enriched glycerolipids, which were recovered at 98.9 ± 1.0% purity (fractions 1–5). Their early elution was consistent with the low $K_{D}$ value of 0.22 and confirmed that the second solvent system efficiently separated remaining glycerolipid material from the pigment region. Chlorophyll c then eluted as a distinct fraction (fractions 9–18, Figure 5) with a purity of 96.9 ± 3.0%, reflecting its intermediate $K_{D}$ value and improved separation from fucoxanthin.

**Figure 6:**
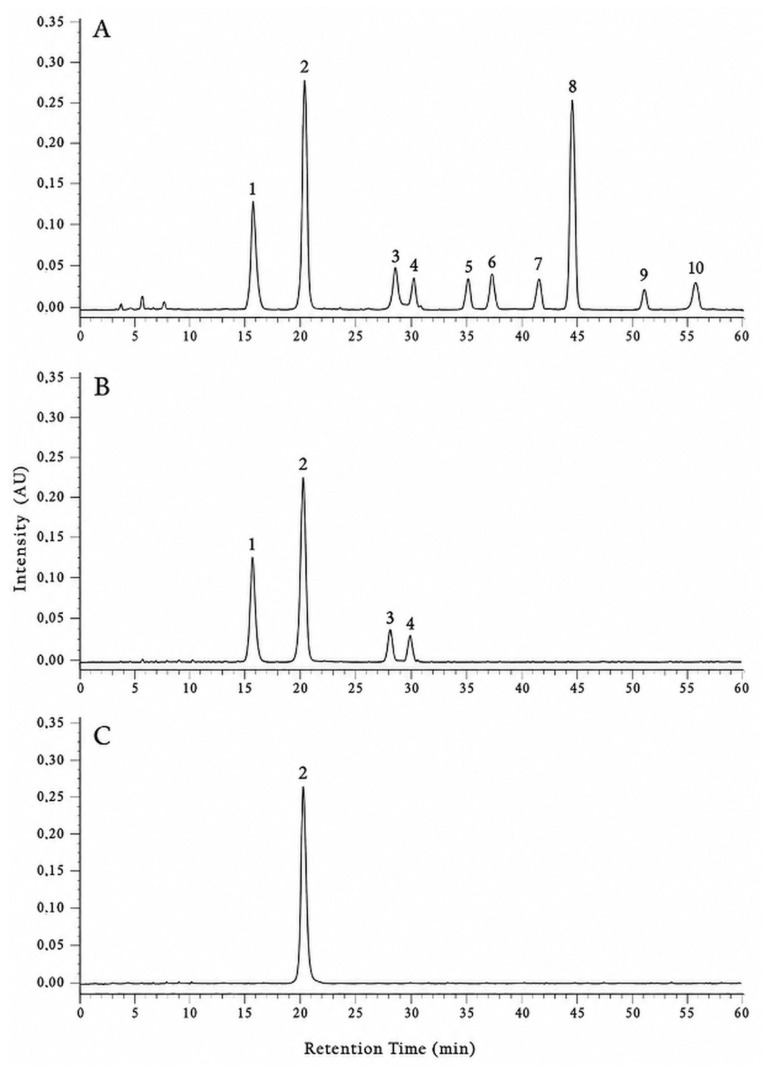
High-performance liquid chromatography with diode-array detection (HPLC–DAD) chromatograms demonstrating the reduction in pigment complexity during purification of fucoxanthin from *Phaeodactylum tricornutum*. Chromatograms correspond to (**A**) the crude lipophilic extract obtained after solid–liquid–liquid extraction (SLLE), (**B**) the fucoxanthin-enriched intermediate fraction obtained after the first CPC run, and (**C**) the purified fucoxanthin fraction obtained after the second CPC run. Signals were extracted at the pigment-specific absorption maxima (λmax) determined from the DAD spectra within the recorded wavelength range (from 350 to 750 nm). Peak identities: chlorophyll c (1), fucoxanthin (2), diadinoxanthin (3), unidentified compound (4), lutein (5), zeaxanthin (6), chlorophyll b (7), chlorophyll a (8), unidentified compound (9), and β-carotene (10).

As shown in Figure 5, the central region of the second CPC chromatogram was able to recover fucoxanthin with 99.4 ± 1.0% purity. The corresponding HPLC profiles confirm the stepwise removal of pigments (Figure 6). In the crude lipophilic extract, fucoxanthin co-occurred with chlorophyll c eluting directly before it and with diadinoxanthin and other minor xanthophylls eluting shortly after it; additional later-eluting hydrophobic pigments, including chlorophyll and β-carotene, were also present (Figure 6A). After the first CPC run, the fucoxanthin-enriched intermediate was strongly simplified: the late-eluting hydrophobic pigment region was largely removed, while chlorophyll c and diadinoxanthin remained the main neighbouring impurities around fucoxanthin (Figure 6B). After the second CPC run, these adjacent pigments were no longer detected in the collected fucoxanthin fraction, which was dominated by a single fucoxanthin peak (Figure 6C). This confirms that the second CPC solvent system resolved the remaining chlorophyll c–fucoxanthin–diadinoxanthin overlap and produced a highly purified fucoxanthin fraction.

Given the selectivity of UV–Vis HPLC detection for chromophoric constituents, complementary high-performance thin-layer chromatography (HPTLC)-based glycerolipid profiling was performed to assess potential residual lipid contaminants not reflected in UV/Vis-HPLC purity estimate (Supplementary Material, Figure S3). Glycerolipids were detected in the crude lipophilic extract and were mainly recovered in the EPA-enriched glycerolipid fractions, whereas no glycerolipid bands were observed in the final fucoxanthin fraction. These results exclude detectable glycerolipid carry-over as a source of apparent HPLC-based purity overestimation and confirm that the second CPC run improved both fucoxanthin chromatographic purity and overall fraction composition. Later fractions from the second run contained a diadinoxanthin-rich fraction with a purity of 82.3 ± 3.0%, followed by a final unresolved minor-component fraction. The later elution of diadinoxanthin was consistent with its higher $K_{D}$ value of approximately 2.00. Although diadinoxanthin was not purified to the same degree as fucoxanthin or chlorophyll c, its recovery as a distinct enriched fraction further demonstrates that the second CPC system improved separation within the carotenoid region.

The final fucoxanthin purity obtained in this work, 99.4 ± 1.0%, is comparable to the highest purities reported for multi-step chromatographic workflows and higher than values typically obtained in single-step separations. However, the significance of the second run is the final purity achieved. More importantly, it shows that the limitation observed during the first CPC run was a selectivity limitation within a narrow pigment cluster. Once the crude extract had been simplified by the first run, the second solvent system provided sufficient partition spacing to resolve chlorophyll c, fucoxanthin, and diadinoxanthin into separate fractions. Thus, the two CPC runs acted as complementary separation stages.

### 2.3. Overall Process Evaluation

The present work extends existing *P. tricornutum* biorefinery concepts by linking wet-biomass fractionation with CPC-based refinement of the resulting lipophilic extract. Previous studies have established *P. tricornutum* as a relevant source of fucoxanthin and EPA [5,6,57,82], demonstrated stepwise recovery of fucoxanthin, EPA, and chrysolaminarin from the same biomass [30], explored solvent-based fractionation of wet *P. tricornutum* biomass [25], and applied CPC/HPCCC or related chromatographic strategies for algal pigment purification [34,35,36,73,81]. In this context, the SLLE–CPC workflow developed here applies liquid–liquid separation at two complementary levels: first, as a fractionating extraction step for wet, disrupted *P. tricornutum* biomass, thereby producing primary product streams, and second, as a preparative purification strategy to refine the lipophilic stream into purified targets and candidate co-products. Table 2 therefore shows that the lipophilic stream can be divided into fractions with different refinement needs and potential applications. Fucoxanthin represents the high-value pigment fraction for which additional polishing is justified, whereas EPA-enriched glycerolipids form a second major product class with potential relevance for nutraceutical, functional food, or aquafeed applications [6,8,57]. The remaining pigment, antioxidant, and neutral-lipid fractions broaden the product spectrum and may serve as enriched blends or intermediates for further upgrading, depending on product specifications and regulatory requirements [11,17,37,38,39,62,72].

Importantly, the lipophilic product portfolio represents only one branch of the overall workflow. The aqueous, interfacial, and insoluble streams generated during SLLE extend the same valorisation logic to non-lipophilic biomass components. Previous studies on *P. tricornutum* support the relevance of carbohydrate-rich, protein-containing, amphiphilic, and structurally insoluble biomass components for future functional or material applications [10,11,64,71]. Their final value will require additional compositional, functional, stability, and safety assessment, but their recovery as separate streams provides a clear basis for subsequent product-oriented development.

From a process-development perspective, the workflow should be viewed in relation to established extraction and preparative liquid–liquid separation technologies. Solid–liquid solvent extraction is widely used in natural-product and food-processing industries, where scale-up is governed by solvent to feed ratio, residence time, mixing, solids handling, phase separation, and solvent recovery [83,84]. For microalgae, Halim et al. [23], Law et al. [24,45] and König-Mattern et al. [25] have shown that wet-biomass extraction and biphasic fractionation can support co-product-oriented processing while avoiding drying-intensive routes. The present SLLE step follows this technical direction but adds the specific challenge of disrupted wet *P. tricornutum* biomass, where interfacial material, solvent availability, and multiphase separation become central process parameters [24,45,52,53].

CPC provides a complementary DSP platform because support-free LLC has already been developed beyond analytical scale. Published pilot- and process-scale LLC reports provide an order-of-magnitude benchmark for scale-up, with industrial CPC rotor designs up to 25 L and large-scale CCC examples covering approximately 0.08–6.6 kg crude extract feed day^−1^, depending on sample solubility, phase-system hydrodynamics, separation selectivity, and operating mode [85,86]. Reported solvent demand varies widely, approximately 76–1460 L kg crude extract^−1^, highlighting that solvent recovery/recycling, loading capacity, cycle time, productivity, and repeated operation would need to be evaluated specifically for the present SLLE–CPC workflow before economic feasibility can be assessed [32,33,85,86,87].

In natural-product processing, CPC/CCC has been widely applied for the purification of specialised metabolites, as reviewed by Pauli et al. and Friesen et al. [75,88]. However, these applications are often target-oriented, focusing on the isolation of one compound or a narrow compound family. Similarly, most algal CPC/HPCCC studies have focused on selected pigments, particularly fucoxanthin or other carotenoids [34,35,36,73,81]. The present workflow therefore uses CPC in a broader process role: as a refinement step for the SLLE-derived lipophilic stream within a multiproduct algal DSP sequence. This distinction is important for further development. Scale-up would not only require larger CPC rotor volume, but also control of stationary-phase retention, loading capacity, peak spacing, solvent consumption, cycle time, and fraction pooling under repeated operation. The close partition behaviour of neighbouring pigments in the chlorophyll *c*–fucoxanthin–diadinoxanthin region shows that product quality at larger scale will depend strongly on solvent-system selectivity. Future work could therefore investigate expanded solvent-system screening, heart-cutting, recycling CPC, two-dimensional CPC strategies, and repeated loading experiments under preparative conditions, while also extending it to other microalgal species owing to the high flexibility of the method.

The modularity of the SLLE–CPC workflow provides clear development levers for such optimisation. The SLLE step can be improved through staged extraction, enhanced phase disengagement, control of interfacial material, and solvent recycling. The CPC part can be intensified selectively for the lipophilic fractions that justify chromatographic refinement. This flexibility is relevant for future techno-economic assessment, since the feasibility of *P. tricornutum* fucoxanthin/EPA production is strongly influenced by cultivation conditions, energy demand, solvent management, process scale, and product value [57,82]. Thus, the scale-up relevance of the workflow lies not in transferring the laboratory procedure unchanged, but in providing a modular process concept in which extraction, phase separation, chromatographic selectivity, and product purity can be optimised according to product demand.

Overall, the SLLE–CPC workflow advances *P. tricornutum* DSP by connecting wet-biomass fractionating extraction, lipophilic-stream refinement, and co-product-oriented fraction recovery within one coherent liquid–liquid separation concept. Together with the product portfolio shown in this study, this provides a scale-up-oriented basis for multiproduct microalgal bioprocess development and supports the transition from target-focused extraction toward an integrated biorefinery approach.

## 3. Materials and Methods

The experimental workflow followed the process sequence shown in Figure 1. Briefly, concentrated *P. tricornutum* biomass was diluted, mechanically disrupted, and subjected to SLLE. After centrifugation-induced phase separation, the lipophilic phase was processed by two-stage CPC, whereas the aqueous, interfacial, and insoluble fractions were analysed as separate product-relevant streams.

### 3.1. Chemicals

All solvents, HPTLC plates (silica gel 60 F_254_, 20 × 10 cm, glass-backed), copper (II) sulphate heptahydrate, potassium chloride, orthophosphoric acid (85%, *w*/*w*), sulfuric acid (96%, *w*/*w*), hydrochloric acid (37%, *w*/*w*), gallic acid, Folin–Ciocalteu (FC) reagent, D (+)-glucose, bovine serum albumin (fraction V), Brilliant Blue G-250, anthrone reagent, and sodium carbonate (Na_2_CO_3_) were purchased from Carl Roth (Karlsruhe, Germany). Primulin, pigment standards (chlorophyll a, chlorophyll c, diadinoxanthin, violaxanthin, lutein, zeaxanthin, β-carotene), lipid-class standards (phosphatidylcholine (PC), phosphatidylethanolamine (PE), phosphatidylinositol (PI), phosphatidylserine (PS), phosphatidylglycerol (PG), monogalactosyldiacylglycerol (MGDG), digalactosyldiacylglycerol (DGDG), sulfoquinovosyldiacylglycerol (SQDG), diacylglyceryltrimethylhomoserin (DGTS), eicosapentaenoic acid (as free fatty acid), triolein, diolein, monoolein as TAG, DAG and MAG and a 37-component fatty acid methyl ester (FAME) mix (Supelco) were purchased from Merck (Darmstadt, Germany). Trans-10-heptadecenoic acid (C17:1) was obtained from Biomol GmbH (Hamburg, Germany).

### 3.2. Biomass Origin and Pretreatment

*P. tricornutum* was produced photoautotrophically in horizontal tubular glass photobioreactors by Simris Group AB (Hammenhög, Sweden). Frozen *P. tricornutum* biomass with an initial DW content of 18.4% was thawed and diluted with demineralized water to 10% DW. Cell disruption was performed using a stirred ball mill (MiniZeta 03, Netzsch, Selb, Germany) for 15 min at a circumferential speed of 10.5 m s^−1^, employing 0.9 mm ZrO_2_ beads at a constant filling degree of 65% (*v*/*v*). Disruption efficiency was evaluated by light microscopy, and only biomass exhibiting a disintegration degree of ≥95% was used for subsequent processing. For analytical purposes, defined amounts of intact biomass were transferred into 2 mL safe-lock tubes (Eppendorf, Hamburg, Germany), mixed with approximately 0.5 mL of 1 mm and 0.3 mL of 0.1 mm glass beads, and processed in a vibration mill (MM500, Retsch, Haan, Germany) at 35 Hz for 20 min. Following cell disruption, target compounds were extracted sequentially (five cycles), each followed by centrifugation at 15,000× *g* for 2 min, and the resulting supernatants were pooled. Lipophilic compounds were extracted using ice-chilled chloroform:methanol (2:1, *v*/*v*), with vortexing for 2 min per cycle. In separate experiments, proteins and carbohydrates were extracted via alkaline treatment using 0.5 M sodium hydroxide (80 °C, 10 min extraction per cycle) in an orbital thermoshaker (Matrix Orbital, IKA, Staufen, Germany). Each cycle was followed by centrifugation at 15,000× *g* for 2 min, and the supernatants were pooled. Total polyphenolic compounds were extracted using ethanolic 0.5 M sodium hydroxide (50%, *v*/*v*) at 50 °C for 10 min per cycle in the same thermoshaker.

### 3.3. Analytical Methods

Unless stated otherwise, analytical measurements were performed in triplicate as technical replicates. For process-level reproducibility, the SLLE DoE experiments were performed as independent extraction experiments: the corner-point experiments were carried out in duplicate, and the centre point was performed 8 times (32 runs in total). The optimised SLLE was subsequently repeated in three independent experiments. CPC separations used for product purity and recovery evaluation were conducted as three independent runs for each CPC separation step. Reported values are expressed as mean ± standard deviation of independent experimental repetitions, while analytical triplicates were used to determine the value for each individual experiment.

#### 3.3.1. Dry Matter and Ash Content

The DW of the biomass was determined gravimetrically using a moisture analyser (MB35, OHAUS^®^, Parsippany, NJ, USA). Approximately 2 g of biomass sample was dried at 105 °C to constant weight. Extracts and CPC fractions were transferred into pre-weighed glass tubes and dried at 105 °C for 24 h in a drying cabinet. Where necessary, samples were first concentrated by rotary evaporation (Hei-VAP Core Handlift, Heidolph, Schwabach, Germany), re-dissolved in a reduced volume, and then transferred into pre-weighed glass tubes. After cooling in a desiccator, samples were reweighed, and DW was calculated from the mass difference. Ash content was determined gravimetrically using ceramic crucibles. A volume of 5 mL of sample was pipetted directly into pre-weighed ceramic crucibles without subsequent washing or rinsing. The samples were dried at 105 °C for 72 h to remove water, cooled to room temperature in a desiccator, and weighed. The crucibles containing the dried residue were then transferred to a muffle furnace and incinerated at 550 °C for 3 h. After incineration, the crucibles were cooled to room temperature in a desiccator and weighed again to determine the ash content.

#### 3.3.2. Glycerolipid Analysis

Lipid classes were quantified by HPTLC as described previously with minor modifications [89]. Samples were applied using an automatic TLC sampler (ATS 4, CAMAG, Muttenz, Switzerland), developed to a migration distance of 85 mm in an automatic developing chamber (ADC 2, CAMAG, Muttenz, Switzerland), derivatized with primulin (0.05%, *w*/*v* in acetone), and detected at 366 nm using a TLC scanner (TLC Scanner 4, CAMAG, Muttenz, Switzerland). Neutral lipids were separated using n-hexane/diethyl ether/acetic acid (70:30:1, *v*/*v*/*v*). Glycolipids and phospholipids were separated using methyl acetate/isopropanol/chloroform/methanol/0.25% aqueous KCl at 25:25:25:10:2 and 25:25:25:10:4.35 (*v*/*v*/*v*/*v*/*v*), respectively. Quantification was based on densitometric peak areas using external calibration with authentic lipid class standards.

#### 3.3.3. Fatty Acid Analysis

Fatty acids were determined in biomass, extracts, and CPC fractions. For lipid class-specific analysis, HPTLC separation was performed prior to transesterification, and the corresponding bands were scraped and subjected to further analysis. In all cases, samples were transesterified by adding 3 mL of methanol and 1 mL of 12 M HCl, together with a known amount of internal standard (C17:1). Transesterification was carried out for 1 h at 95 °C in a water bath. The resulting fatty acid methyl esters (FAMEs) were extracted with n-hexane, filtered through 0.22 µm polytetrafluoroethylene (PTFE) filters, and analysed by gas chromatography–mass spectrometry (GC–MS; HP 6890 GC coupled to a 5972 MSD, Agilent Technologies, Santa Clara, CA, USA). Separation was performed on a BPX-70 column (30 m × 0.32 mm, 0.25 µm; SGE Analytical Science, Ringwood, Australia) using the following temperature program: 50 °C for 1 min, increased at 8 °C min^−1^ to 170 °C with a 2 min hold, followed by 1 °C min^−1^ to 250 °C with a 10 min hold. Helium was used as carrier gas at a flow rate of 0.9 mL min^−1^. FAMEs were identified based on retention times of a 37-component FAME standard mixture and by comparison with mass spectra from the National Institute of Standards and Technology (NIST) database. Quantification was performed by external calibration.

#### 3.3.4. Pigment Analysis

Chlorophylls and carotenoids were analysed in biomass, extracts, and CPC fractions by HPLC with diode array detection (DAD) following the method described by Grewe (2009) with minor modifications [90]. The system consisted of a D-6000 interface (Hitachi, Tokyo, Japan), an ERC-3113 degasser (Erma, Tokyo, Japan), an L-6200A pump (Merck Hitachi, Tokyo, Japan), an AS-4000A autosampler (Merck Hitachi, Tokyo, Japan), and an L-7450A diode array detector (Merck Hitachi, Tokyo, Japan). A column thermostat (Jetstream 2 Plus, JASCO, Tokyo, Japan) was used in combination with a ProntoSIL 300-5-C30 column (250 × 4.6 mm, 5 µm; Bischoff Chromatography, Leonberg, Germany), maintained at 25 °C. Samples were filtered through 0.22 µm PTFE syringe filters prior to injection. Where necessary, samples were concentrated by rotary evaporation (Hei-VAP Core Handlift, Heidolph, Schwabach, Germany) prior to analysis. Separation was performed using methanol/water (75:25, *v*/*v*) as solvent A and ethyl acetate as solvent B at a flow rate of 0.6 mL min^−1^. The gradient program was as follows: 70% A to 45% A over 25 min, followed by a decrease to 10% A over 25 min, holding for 10 min, and returning to initial conditions over 10 min. Spectra were recorded from 300 to 750 nm. Pigments were identified based on retention behaviour and absorption spectra and quantified at individual λmax of each pigment by external calibration using authentic standards.

#### 3.3.5. Protein, Carbohydrate and Polyphenolic Analysis

The colorimetric assays were performed using modified protocols adapted from previously described methods to the available laboratory equipment, reagent volumes, and 96-well microplate format. Protein content was determined using a modified Bradford assay [91]. In brief, bovine serum albumin was used as standard, and the Bradford reagent consisted of 20 mg Coomassie Brilliant Blue G-250 dissolved in 25 mL ethanol and 50 mL of 85% phosphoric acid and diluted to 500 mL with deionized water. Sample aliquots (20 µL) were mixed with 200 µL of Bradford reagent and incubated for 5 min at room temperature, and absorbance was measured at 595 nm. Total carbohydrate content was determined using a modified anthrone–sulfuric acid assay [92,93]. In brief, D(+)-glucose was used as standard, and the anthrone reagent consisted of 0.2% (*w*/*v*) anthrone in 96% H_2_SO_4_. Samples were reacted with the anthrone reagent, heated at 100 °C for 15 min, cooled to room temperature, and measured at 625 nm. Total phenolic content was determined using a modified Folin–Ciocalteu (FC) assay [94,95]. In brief, the FC reagent was diluted 1:10 (*v*/*v*) with water. Samples were reacted with the diluted FC reagent and Na_2_CO_3_ (7.5% *w*/*v*), incubated at 42 °C for 30 min, and measured at 720 nm. All spectrophotometric measurements were performed in 96-well plates (F-bottom) using a microplate reader (Infinite M200, Tecan, Männedorf, Switzerland).

### 3.4. Optimisation of the Solid–Liquid–Liquid Extraction

An SLLE procedure based on a biphasic ethyl acetate–n-butanol–water system (3:2:5, *v*/*v*/*v*) was optimised using RSM with a D-optimal quadratic design comprising 32 experimental runs. This extraction step represented the primary fractionation stage of the overall workflow shown in Figure 1 and was designed to distribute disrupted biomass components into chemically distinct lipophilic, aqueous, interfacial, and insoluble fractions. The independent variables were extraction time and biomass content. The lower and upper levels of the investigated variables were selected to define a technically feasible operating window for wet-biomass SLLE. The extraction-time range of 0.5–5.0 h was chosen to include short, process-relevant contact times as well as longer extraction periods allowing extended mass transfer. The biomass-content range of 1–10% (*w*/*w*) was selected to evaluate the effect of increasing solids loading while maintaining adequate mixing, solvent contact, and phase separation. Extraction temperature was fixed at 25 °C to avoid additional thermal stress, and all extraction flasks were covered with aluminium foil throughout the extraction to minimise light exposure and reduce photo-induced degradation of fucoxanthin and EPA-containing lipids. No inert atmosphere or antioxidant was applied during the SLLE optimisation. The D-optimal design was generated within these predefined factor ranges using a quadratic model. The final experimental plan consisted of 12 unique non-centre design points, each performed in duplicate, resulting in 24 non-centre runs, and one centre-point condition, corresponding to 2.75 h and 5.5% (*w*/*w*) biomass content, repeated eight times to estimate pure experimental error. Thus, the complete design comprised 32 experimental runs. The actual extraction-time levels included in the design were 0.5, 2.0, 2.75, 3.5, and 5.0 h, while the actual biomass-content levels were 1, 4, 5.5, 7, and 10% (*w*/*w*). The selected design had a G-efficiency of 61.01% and a condition number of 3.30.

Extractions were conducted in 50 mL Erlenmeyer flasks, covered in alufoil, with a total working volume of 20 mL under magnetic stirring at 150 rpm using a 3 cm stir bar. Following extraction, samples were centrifuged at 4643× *g* for 2 h to achieve adequate phase separation.

The recovery of the target compound *i* was calculated according to Equation (1):

(1) $$R_{i,\;extraction}\left(\%\right)=\frac{m_{i,\;\;extract}}{m_{i,biomass}}\times 100$$

where $m_{i,extract}$ is the mass of compound *i* recovered in the extract phase, and $m_{i,biomass}$ is the initial mass of compound *i* in the biomass. In this study, *i* refers either to EPA or fucoxanthin. The recovery is expressed as the percentage of the initial compound content recovered from the biomass.

The extraction yield was determined using Equation (2):

(2) $$Y_{extract}=\frac{m_{j,extract}}{m_{biomass}}$$

where $m_{j,extract}$ is the DW of the extracted fraction *j*, and $m_{biomass}$ is the DW of the processed biomass. In this case, *j* represents either the aqueous fraction or the lipophilic fraction. The extraction yield is therefore expressed as grams of extract dry matter per gram of biomass dry matter ($g_{extract\;DW}\;g_{biomass\;DW}^{-1}$). Experimental data were fitted to a second-order polynomial model including linear, quadratic, and interaction terms. Model adequacy was assessed by analysis of variance (ANOVA), R^2^, adjusted R^2^, and lack-of-fit testing. The experimental design and response surface modelling were implemented in Python (v3.11). For optimisation, two different decision frameworks were applied. First, a recovery-driven optimisation was used to identify the analytical maximum-recovery point within the model domain. Second, a process-oriented optimisation was used to identify an operating window more relevant for scale-up. In the latter case, the optimisation was performed as a two-stage procedure. A lexicographic feasibility screen was first applied to retain only points fulfilling minimum acceptable performance criteria, namely fucoxanthin recovery ≥ 90%, EPA recovery ≥ 70%, lipophilic fraction ≥ 0.165 g g^−1^ DW, hydrophilic fraction ≥ 0.295 g g^−1^ DW, and pellet fraction ≤ 0.43 g g^−1^ DW. The remaining feasible points were then ranked using a global desirability function based on the weighted geometric mean of individual desirabilities, following the Derringer–Suich framework. To ensure that the optimisation reflected an optimal performance rather than analytical extraction exhaustiveness, the four principal product responses were expressed as throughput-adjusted productivity terms (Supplementary Material, Table S3). The selected operating point was then experimentally validated under the chosen process conditions.

### 3.5. CPC

As indicated in Figure 1, CPC was applied only to the lipophilic fraction obtained after biphasic extraction. The CPC workflow was designed as a two-stage separation sequence, in which the first run served as a broad capture and pre-fractionation step, while the second run was used to polish the fucoxanthin-enriched intermediate. All solvents used in the CPC experiments were of pro analysis (p.a.) grade. CPC separations were performed using an SCPC-250 system (Armen Instrument, Paris, France) equipped with an HPLC pump and a photodiode array (PDA)-UV/Vis detector (AlphaCrom AG, Rheinfelden, Switzerland), as well as an FC-260 fraction collector (Teledyne Technologies, Thousand Oaks, CA, USA). The rotor column, made of stainless steel, consisted of 20 discs each containing 90 twin cells, resulting in a total of 1800 cells. The measured rotor volume was 247 mL. Sample injection was performed using a 10 mL sample loop. $S_{f}$ was determined volumetrically and monitored throughout each run, while column bleeding was recorded during the separations. System control and data acquisition were performed using the PrepCon 5 control and data handling system (Sykam GmbH, Eresing, Germany), enabling real-time monitoring and automated method execution.

#### 3.5.1. Solvent System Selection

Biphasic solvent systems were selected based on determination of $K_{D}$ values (Equation (3)) using the shake-flask partitioning method. Approximately 2 mg of extract was equilibrated with 1 mL of each pre-equilibrated upper and lower phase. After mixing and phase separation, aliquots from both phases were analysed by HPLC–DAD, GC–MS and HPTLC as described above. Distribution coefficients were calculated as

(3) $$K_{d}=\frac{c_{\mathrm{upper}\;\mathrm{phase}}}{c_{\mathrm{lower}\;\mathrm{phase}}}$$

where c is expressed as the concentration of the examined compounds in the phases in mg L^−1^.

#### 3.5.2. Separation via CPC

A hexane/ethyl acetate/methanol/water (8:2:8:2, *v*/*v*/*v*/*v*) system was employed for primary fractionation of the crude lipophilic product stream. The upper phase was used as the stationary phase in descending mode. The rotor was filled at 20 mL min^−1^ and 55.3× *g* followed by acceleration to 717× *g* for 10 min. The mobile phase (lower phase) was then introduced at 5 mL min^−1^ to establish hydrodynamic equilibrium. Stationary phase retention ($S_{f}$, Equation (4)) was determined volumetrically and expressed as the volumetric ratio of the stationary phase ($V_{\mathrm{stationary}\;\mathrm{phase}}$, mL) to the whole rotor volume ($V_{\mathrm{rotor}}$, mL).

(4) $$S_{f}=\frac{V_{\mathrm{stationary}\;\mathrm{phase}}}{V_{\mathrm{rotor}}}$$

The column bleeding did not exceed 7 mL per run. Sample loading was 200 mg of crude extract per 247 mL rotor volume. Fractions were collected on a volume basis (10 mL per fraction). Collected fractions were analysed by HPLC, HPTLC, and GC–MS as described above. In the first CPC run, after collection of fraction n°30, the operating mode was switched from descending to ascending, while the same flow rate was applied continuously throughout the separation.

Fucoxanthin-enriched fractions obtained from the first CPC separations were collected, and the solvents were removed by rotary evaporation (Hei-VAP Core Handlift, Heidolph, Schwabach, Germany). The fucoxanthin-enriched fraction (200 mg) was reconstituted in the lower phase of the second solvent system, which contained n-heptane/ethyl acetate/ethanol/water, 5:5:5.4:4.6, *v*/*v*/*v*/*v*) and was subjected to a second CPC separation. In the second CPC run, the rotor was filled similarly at 20 mL min^−1^ and 55× *g* in descending mode, followed by acceleration to 717× *g*. The mobile phase (lower phase) was then introduced at 4 mL min^−1^ to establish hydrodynamic equilibrium. Stationary phase retention was determined volumetrically. The separation was performed under constant descending mode. Fractions were collected on a volume basis (10 mL per fraction). Column bleeding did not exceed 5 mL per run.

In the experiments, purity (P, Equation (5)) was defined as the proportion of the quantified amount of the target compound relative to the total measured mass of the corresponding collected or pooled fraction:

(5) $$P\left(\%\right)=\frac{m_{i}}{m_{\mathrm{fraction}}}\times 100$$

where $m_{i}$ is the quantified mass of the target compound in the fraction, and $m_{fraction}$ is the total DW of the corresponding pooled fraction, defined gravimetrically. Purity therefore represents the percentage contribution of the target compound within the collected material. For the EPA-enriched glycerolipid fractions the basis was the sum of the measured phospho- and galactolipid classes containing EPA, whereas for the neutral lipid fraction, the basis was the sum of TAG, DAG, MAG and FFA.

### 3.6. Statistical Experimental Analysis

Data processing and visualisation were performed in Python (v3.11.13) using the libraries Pandas (v2.3.3), NumPy (v2.4.6), SciPy (v1.16.x), Matplotlib (v3.10.x), and Seaborn (v0.13.2). SLLE performance was evaluated using response surface methodology (RSM) based on a D-optimal quadratic design. Model coefficients, ANOVA results, and diagnostic parameters are provided in Supplementary Material (Table S1 and S2).

## 4. Conclusions

This study demonstrates a liquid–liquid separation-based DSP workflow for enhanced multiproduct valorisation of wet, disrupted *P. tricornutum* biomass. SLLE with ethyl acetate/n-butanol/water served as an early pre-fractionation step, separating the biomass into lipophilic, aqueous, interfacial, and insoluble fractions while maintaining high recovery of the principal targets. Under the process-oriented setpoint of 1.3 h and 4.25% biomass content, fucoxanthin and EPA recoveries reached 91.3% and 70.0%, respectively, while 16.5% of the initial biomass DW was forwarded to CPC. This reduced the chromatographic load and provided a pre-enriched lipophilic stream with approximately 5.3-fold fucoxanthin enrichment and at least 4.2-fold EPA enrichment.

Staged CPC further converted this lipophilic stream into multiple product-relevant fractions rather than a single purified compound. The workflow yielded high-purity fucoxanthin (99.4 ± 1.0%), EPA-enriched glycerolipids (up to 99.9 ± 0.5%), and additional pigment, antioxidant, and lipid fractions, resulting in twelve distinct output fractions from the same biomass input. The process therefore supports a broader biorefinery strategy in which co-extracted compounds are retained as valuable product streams instead of being treated as impurities or residual biomass.

Overall, the combination of scalable SLLE with preparative CPC provides a practical foundation for multiproduct DSP development in *P. tricornutum* and points toward a promising route for advancing microalgal biorefinery strategies.

## Figures and Tables

**Table 1:** Phase distribution, biochemical composition, and mass balance of fractions obtained by solid–liquid–liquid extraction of disintegrated *Phaeodactylum tricornutum* using an ethyl acetate/n-butanol/water solvent system (3:2:5, *v*/*v*/*v*). Extractions were performed for 1.3 h at a biomass loading of 4.25% (*w*/*w*, dry weight (DW)). Distribution values are expressed as milligrams per gram of dry biomass (mg g*_biomass_*^−1^ DW), representing the amount of recovered component in each fraction from the biomass, whereas fraction compositions are expressed as milligrams per gram of dry fraction (mg g*_fraction_*^−1^ DW).

Fraction	Fraction Phase Yield [*v*/*v*]	Component	mg g_biomass_^−1^ DW	mg g_fraction_^−1^ DW
Lipophilic fraction	0.45 ± 0.02	Glycerolipids	119 ± 8	719 ± 66
		Chlorophyll c	3.00 ± 0.20	18.2 ± 1.7
		Fucoxanthin	14.1 ± 0.9	85.3 ± 7.8
		Diadinoxanthin	0.80 ± 0.10	4.8 ± 0.7
		Lutein	0.2 ± 0.0	1.20 ± 0.10
		Zeaxanthin	0.70 ± 0.10	4.2 ± 0.7
		Chlorophyll a	13.6 ± 0.9	82.3 ± 7.6
		β-Carotene	1.20 ± 0.10	7.3 ± 0.8
		Lipophilic polyphenols	2.10 ± 0.20	13 ± 1.3
		Unidentified lipophilic compounds	10.7 ± 0.6	64.8 ± 5.8
		Total lipophilic extract	165 ± 11	
Interfacial fraction	0.05 ± 0.01	Glycerolipids	30.1 ± 1.9	266 ± 23
		Interface-associated proteins	39.9 ± 2.5	353 ± 31
		Non-glycerolipid-like lipophilic compounds	9.0 ± 0.6	79.5 ± 6.9
		Unidentified interfacial material	34.2 ± 2.1	302 ± 26
		Total interfacial fraction	113 ± 7	
Aqueous fraction	0.39 ± 0.02	Soluble proteins and peptides	50.0 ± 3.0	165 ± 14
		Water-soluble carbohydrates	165 ± 10	544 ± 47
		Ash	40.0 ± 2.4	132 ± 11
		Hydrophilic polyphenols	1.00 ± 0.10	3.3 ± 0.4
		Unidentified hydrophilic metabolites	48.4 ± 2.9	160 ± 14
		Total aqueous extract	303 ± 18	
Insoluble residue	0.11 ± 0.02	Polysaccharides	150 ± 9	359 ± 31
		Proteins	110 ± 7	263 ± 22
		Ash	55.0 ± 3.3	132 ± 11
		Residual lipophilic compounds	25.0 ± 1.5	59.8 ± 5.1
		Unidentified insoluble material	78.2 ± 4.7	187 ± 16
		Total insoluble residue	418 ± 25	

**Table 2:** Compositionally defined product fractions obtained from the lipophilic extract of *Phaeodactylum tricornutum* by staged centrifugal partition chromatography (CPC), including purity and proposed applications.

Product	CPC Step/ Fractions	Purity * [%]	Most Plausible Usage
Polyphenolics	Run 1, n°1–3	68.7 ± 4.0	Antioxidant-rich cosmetic or nutraceutical blend
EPA-enriched glycerolipids	Run 1, n°4–16	99.9 ± 0.5	Omega-3 lipid ingredient for nutraceuticals, functional foods, aquafeed
	Run 2, n°1–6	98.9 ± 1.0	
Fucoxanthin	Run 1, n°17–29	45.4 ± 3.5	High-value nutraceutical, cosmeceutical, bioactive ingredient
	Run 2, n°19–29	99.4 ± 1.0	
β-Carotene	Run 1, n°31–32	73.9 ± 3.0	Natural colourant, antioxidant blend, upgrade feed
Neutral lipids	Run 1, n°34–36	97.4 ± 1.5	oleochemicals, feed
Chlorophyll a	Run 1, n°37–42	95.1 ± 0.7	Specialty pigment, cosmetic colour, analytical/research use
Carotenoid mixture	Run 1, n°43–48	34.1 ± 2.0	Blended pigment, colourant/feed additive
Chlorophyll c	Run 2, n°9–18	96.9 ± 3.0	Specialty pigment, cosmetic colour, analytical/research use
Diadinoxanthin	Run 2, n°30–41	82.3 ± 3.0	Secondary carotenoid product, antioxidant blend

* Table note: Purity values were calculated as the quantified target compound or target compound class relative to the gravimetrically determined dry weight of the corresponding pooled CPC fractions. The analytical method used depended on the respective product group. Pigment fractions (chlorophylls and carotenoids) were quantified by high-performance liquid chromatography with diode-array detection (HPLC–DAD) and, where relevant, additionally assessed by high-performance thin-layer chromatography (HPTLC) to exclude co-eluting lipid classes. Lipid fractions, including eicosapentaenoic acid (EPA)-enriched glycerolipids and neutral lipids, were assessed by HPTLC-based lipid-class analysis, with gas chromatography–mass spectrometry (GC–MS) of fatty acid methyl esters (FAMEs) used where applicable for fatty acid confirmation, and HPLC–DAD to verify the absence of co-eluting pigments. The polyphenolic fraction was assessed by the Folin–Ciocalteu assay and HPLC–DAD.

## Data Availability

The original contributions presented in the study are included in the article; further inquiries can be directed to the corresponding authors.

## Supplementary Materials

The following supporting information can be downloaded at: https://www.mdpi.com/article/10.3390/md24070242/s1. Supplementary Figure S1: Response surface plots illustrating the influence of extraction time and biomass content (dry-weight (DW) basis) on the emulsion/interlayer fraction (g g^−1^ biomass DW) during solid–liquid–liquid extraction (SLLE) of disrupted wet Phaeodactylum tricornutum biomass using ethyl acetate/n-butanol/water (3:2:5, *v*/*v*/*v*) at 25 °C (disintegration degree > 95%). Supplementary Figure S2: Response surface plots illustrating the influence of extraction time and biomass content (dry-weight (DW) basis) on the residual pellet fraction (g g^−1^ biomass DW) during solid–liquid–liquid extraction (SLLE) of disrupted wet *Phaeodactylum tricornutum* biomass using ethyl acetate/n-butanol/water (3:2:5, *v*/*v*/*v*) at 25 °C (disintegration degree > 95%). Supplementary Figure S3: Top-view densitometric scan of high-performance thin-layer chromatography (HPTLC) analysis of the crude lipophilic extract and purified fucoxanthin fraction. Supplementary Table S1: Goodness-of-fit and validation statistics for the quadratic response surface models describing fucoxanthin recovery, eicosapentaenoic acid (EPA) recovery, and biomass fraction distribution. Supplementary Table S2: Regression coefficients of the quadratic response surface models describing fucoxanthin recovery (%) and eicosapentaenoic acid (EPA) recovery (%), as well as lipophilic, hydrophilic, emulsion, and pellet fractions yields (g g^−1^ dry weight (DW)). Supplementary Table S3. Ten highest-ranked candidate operating conditions for the solid–liquid–liquid extraction of *Phaeodactylum tricornutum* biomass identified by lexicographic feasibility screening followed by weighted multi-response optimization. Predicted responses were calculated from the fitted quadratic response surface models and candidate conditions were ranked using productivity-based criteria for fucoxanthin (Fx) recovery, eicosapentaenoic acid (EPA) recovery, lipophilic fraction, and hydrophilic fraction together with process operability constraints including biomass content, extraction time, residual pellet fraction, and emulsion/interlayer formation. DW: Dry weight.
