# Supplementary material for: Towards Microalgal Biorefinery: Multiproduct Fractionation of Phaeodactylum tricornutum by Liquid–Liquid Techniques
Source: Mar Drugs. 2026 Jul 9;24(7):242. doi: 10.3390/md24070242 (PMC13413208; doi:10.3390/md24070242)
Supplement: Supplementary file 1 [file marinedrugs-24-00242-s001.zip › marinedrugs-4405319-supplementary.pdf]

Supplementary Material – Figures

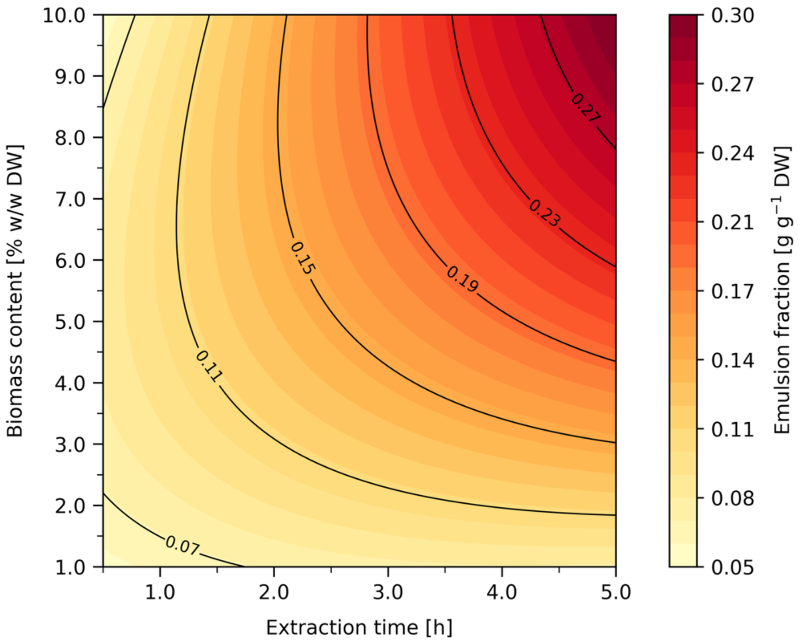

Supplementary Figure. S1. Response surface plots illustrating the influence of extraction time and biomass content (dry-weight (DW) basis) on the emulsion/interlayer fraction yield (g g^{-1} biomass DW) during solid-liquid-liquid extraction (SLLE) of disrupted wet *Phaeodactylum tricornutum* biomass using ethyl acetate–n-butanol–water (3:2:5, v/v/v) at 25 °C (disintegration degree > 95%).

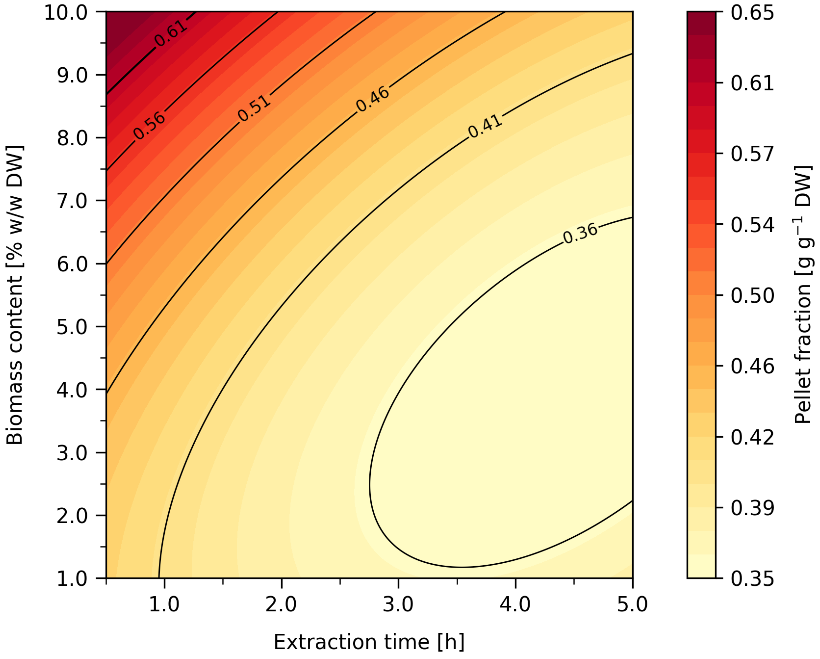

Supplementary Figure S2. Response surface plots illustrating the influence of extraction time and biomass content (dry-weight (DW) basis) on the residual pellet fraction yield (g g^{-1} biomass DW) during solid-liquid-liquid extraction (SLLE) of disrupted wet *Phaeodactylum tricornutum* biomass using ethyl acetate–n-butanol–water (3:2:5, v/v/v) at 25 °C (disintegration degree > 95%).

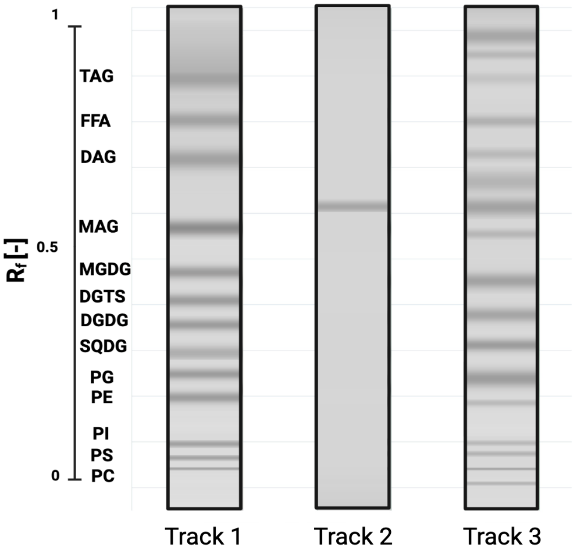

Supplementary Figure S3. Top-view densitometric scan of high-performance thin-layer chromatography (HPTLC) analysis of the crude lipophilic extract and purified fucoxanthin fraction.

Figure Note: High-performance thin-layer chromatography (HPTLC) was performed using a silica gel 60 F_{254} HPTLC plate (10 x 20 cm) and three consecutive solvent developments. The first development employed methyl acetate/isopropanol/chloroform/methanol/0.25% aqueous potassium chloride (KCl), acidified with glacial acetic acid (25:25:25:10:4.35, v/v/v/v/v), followed by development with n-hexane/acetone/isopropanol (80:20:5, v/v/v) and a final development with n-hexane/diethyl ether/glacial acetic acid (70:30:1, v/v/v). The plate was derivatized with the modified copper sulfate reagent consisting of 20 g copper(II) sulfate heptahydrate ($CuSO_4{\cdot}7H_2O$) dissolved in 200 mL methanol and acidified with 8 mL of 96% (w/w) sulfuric acid (H_2SO_4) and 8 mL of 85% (w/w) orthophosphoric acid (H_3PO_4). Densitometric scan was recorded at 520 nm. **Track 1:** Lipid standard mixture (600 ng band^{-1}). From top to bottom: triacylglycerols (TAG), free fatty acids (FFA), diacylglycerols (DAG), monoacylglycerols (MAG), monogalactosyldiacylglycerols (MGDG), diacylglyceryltrimethyl-homoserine (DGTS), digalactosyl-diacylglycerols (DGDG), sulfoquino-vosyldiacylglycerols (SQDG), phosphatidyl-glycerol (PG), phosphatidylethanolamine (PE), phosphatidyl-inositol (PI), phosphatidylserine (PS), and phosphatidylcholine (PC). **Track 2:** Purified final fucoxanthin fraction obtained after two-stage centrifugal partition chromatography (CPC) purification. **Track 3:** Crude lipophilic extract of *Phaeodactylum tricornutum* obtained by solid–liquid–liquid extraction (SLLE) using the ethyl acetate/n-butanol/water (3:2:5, v/v/v) solvent system prior to CPC purification. Rf: Retention factor.

Supplementary Material – Tables

Table S1. Goodness-of-fit and validation statistics for the quadratic response surface models describing fucoxanthin recovery, eicosapentaenoic acid (EPA) recovery, and biomass fraction distribution.

**Response**	**R^2**	**Adj. R^2**	**Q^2**	**Model F-value**	**Model p-value**
Fucoxanthin recovery [%]	0.888	0.866	0.823	41.13	1.54×10^{-11}
EPA recovery [%]	0.782	0.740	0.647	18.65	7.25×10^{-8}
Lipophilic fraction [g g^{-1} DW]	0.934	0.921	0.892	73.20	1.77×10^{-14}
Hydrophilic fraction [g g^{-1} DW]	0.851	0.822	0.723	29.60	5.99×10^{-10}
Emulsion fraction [g g^{-1} DW]	0.915	0.898	0.852	55.76	4.53×10^{-13}
Pellet fraction [g g^{-1} DW]	0.952	0.943	0.920	103.78	2.51×10^{-16}

Table note: R^2 indicates the coefficient of determination, adjusted R^2 (Adj. R^2) indicates the coefficient of determination adjusted for the number of model terms, and Q^2 indicates the predictive ability of the model determined by cross-validation. The model F-value represents the overall analysis-of-variance test statistic for the fitted quadratic model, comparing explained model variance with residual variance. The model p-value indicates the probability of obtaining the observed F-value under the null hypothesis that the model terms do not explain the response; lower p-values therefore indicate stronger statistical evidence for model significance.

Table S2. Regression coefficients of the quadratic response surface models describing fucoxanthin recovery (%) and eicosapentaenoic acid (EPA) recovery (%), as well as lipophilic, hydrophilic, emulsion, and pellet fractions yields (g g^{-1} dry weight (DW)).

**Response**	**Intercept (β_0)**	**x_1**	**x_2**	**x_1x_2**	**x_1^2**	**x_2^2**
Fucoxanthin recovery	93.6616	4.5705	−11.4614	−3.9310	−1.1203	−18.4974
EPA recovery	72.7401	5.7788	−15.9551	−3.5533	−4.6049	−11.3732
Lipophilic fraction	0.1682	0.0008	−0.0632	−0.0041	−0.0392	−0.0041
Hydrophilic fraction	0.2858	0.0045	−0.0645	−0.0044	0.0096	−0.0200
Emulsion fraction	0.1615	0.0681	0.0547	0.0569	−0.0090	−0.0300
Pellet fraction	0.3848	−0.0736	0.0736	−0.0488	0.0379	0.0548

Table note: Functions are coded as follow: extraction time $x_1$and biomass content x_2. Models were fitted as $Y = \beta_0 + \beta_1 x_1 + \beta_2 x_2 + \beta_{12} x_1 x_2 + \beta_{11} x_1^2 + \beta_{22} x_2^2$, where $x_1 = (t - 2.75)/2.25$ with t = extraction time [h] and $x_2 = (B - 5.5)/4.5$with B= biomass content [%, w/w DW]; temperature was maintained constant at 25 °C and therefore not included as a model factor.

Table S3. Ten highest-ranked candidate operating conditions for the solid-liquid-liquid extraction of *Phaeodactylum tricornutum* biomass identified by lexicographic feasibility screening followed by weighted multi-response optimization. Predicted responses were calculated from the fitted quadratic response surface models and candidate conditions were ranked using productivity-based criteria for fucoxanthin (Fx) recovery, eicosapentaenoic acid (EPA) recovery, lipophilic fraction, and hydrophilic fraction together with process operability constraints including biomass content, extraction time, residual pellet fraction, and emulsion/interlayer formation. DW: Dry weight.

Rank	Time [h]	Biomass [DW %]	Fx recovery [%]	EPA recovery [%]	Lipophilic yield [g $g_{biomass}^{-1}$ DW]	Hydrophilic yield [g $g_{biomass}^{-1}$ DW]	Interlayer [g $g_{biomass}^{-1}$ DW]	Pellet [g $g_{biomass}^{-1}$ DW]	Relative score	Fx productivity [mg $g_{suspension}^{-1}$ h^{-1}]	EPA productivity [mg $g_{suspension}^{-1}$ h^{-1}]
**1**	1.30	4.25	91.30	70.02	0.168	0.302	0.107	0.423	100.0	0.448	0.870
**2**	1.35	4.40	91.48	70.04	0.167	0.301	0.109	0.423	99.6	0.447	0.867
**3**	1.25	4.05	91.05	70.04	0.169	0.305	0.104	0.422	99.3	0.442	0.862
**4**	1.40	4.55	91.61	70.03	0.166	0.299	0.112	0.423	99.2	0.447	0.865
**5**	1.30	4.20	91.29	70.10	0.169	0.303	0.106	0.422	99.0	0.442	0.861
**6**	1.35	4.35	91.48	70.13	0.168	0.301	0.109	0.422	98.6	0.442	0.859
**7**	1.25	4.00	91.01	70.11	0.170	0.305	0.103	0.421	98.3	0.437	0.852
**8**	1.40	4.50	91.63	70.12	0.167	0.299	0.111	0.422	98.3	0.442	0.857
**9**	1.30	4.15	91.27	70.18	0.169	0.304	0.106	0.421	98.0	0.437	0.851
**10**	1.45	4.65	91.72	70.08	0.166	0.297	0.114	0.423	97.8	0.441	0.854

Table note: For optimization, two different decision frameworks were applied. First, a recovery-driven optimisation was used to identify the analytical maximum-recovery point within the model domain. Second, a process-oriented optimization was used to identify an optimal operating window. In the latter case, the optimisation was performed as a two-stage procedure. A lexicographic feasibility screen was first applied to retain only points fulfilling minimum acceptable performance criteria, namely fucoxanthin recovery ≥90%, eicosapentaenoic acid (EPA) recovery ≥ 70%, lipophilic fraction ≥ 0.165 g $g_{biomass}^{-1}$ dry weight (DW), hydrophilic fraction ≥ 0.295 g $g_{biomass}^{-1}$ DW, and pellet fraction ≤ 0.43 g $g_{biomass}^{-1}$ DW.

To ensure that the optimisation reflected optimal performance rather than extraction exhaustiveness alone, the principal product responses were expressed as throughput-adjusted productivity terms. Biomass loading was represented by the biomass mass fraction in the slurry, w_{biomass} (g $g_{\mathrm{suspension}}^{-1}$). Fucoxanthin and EPA productivities were defined as

$$P_{\mathrm{Fuco}} = \frac{w_{\mathrm{biomass}} \cdot C_{\mathrm{Fucoxanthin}} \cdot R_{\mathrm{Fucoxanthin}}(t, w_{\mathrm{biomass}})}{t}$$

$$P_{\mathrm{EPA}} = \frac{w_{\mathrm{biomass}} \cdot C_{\mathrm{EPA}} \cdot R_{\mathrm{EPA}}(t, w_{\mathrm{biomass}})}{t}$$

and the bulk-fraction productivities were defined as

$$P_{\mathrm{Lipophilic}} = \frac{w_{\mathrm{biomass}} \cdot Y_{\mathrm{Lipophilic}}(t, w_{\mathrm{biomass}})}{t}$$

$$P_{\mathrm{Hydrophilic}} = \frac{w_{\mathrm{biomass}} \cdot Y_{\mathrm{Hydrophilic}}(t, w_{\mathrm{biomass}})}{t}$$

$$P_{\mathrm{interlayer}} = \frac{w_{\mathrm{biomass}} \cdot Y_{\mathrm{Interlayer}}(t, w_{\mathrm{biomass}})}{t}$$

$$P_{\mathrm{pellet}} = \frac{w_{\mathrm{biomass}} \cdot Y_{\mathrm{Pellet}}(t, w_{\mathrm{biomass}})}{t}$$

where t is extraction time (hour), C_{Fuco} and C_{EPA} are the experimentally determined maximal content of fucoxanthin and EPA in the biomass (mg g^{-1} DW), $R_{\mathrm{Fuco}}(t, w_{\mathrm{biomass}})$ and $R_{\mathrm{EPA}}(t, w_{\mathrm{biomass}})$ are the model-predicted recoveries, and $Y_{\mathrm{Lipophilic}}(t, w_{\mathrm{biomass}})$, $Y_{\mathrm{Hydrophilic}}(t, w_{\mathrm{biomass}})$, $Y_{\mathrm{interlayer}}(t, w_{\mathrm{biomass}})$, $Y_{\mathrm{pellet}}(t, w_{\mathrm{biomass}})$ are the model-predicted yields all expressed as functions of extraction time and biomass mass fraction in mg $g_{\mathrm{biomass}}^{-1}$. In the present study, $C_{\mathrm{Fucoxanthin}} = 15$ mg g^{-1} DW and $C_{\mathrm{EPA}} = 38$ mg g^{-1} DW. With this formulation, P_{Fuco}, P_{EPA}, $P_{\mathrm{Lipophilic}}$, $P_{\mathrm{Hydrophilic}}$, $P_{\mathrm{interlayer}}$, P_{pellet} are expressed as mg $g_{\mathrm{suspension}}^{-1}$ h^{-1}.

Individual desirability functions d_i were calculated according to the Derringer–Suich approach, transforming each response into a dimensionless value between 0 and 1. For responses to be maximized (productivities), desirability increased from 0 at the lower bound to 1 at the target value using a linear scaling function. For responses to be minimized (pellet fraction and emulsion/interlayer), desirability decreased from 1 at the target value to 0 at the upper bound. A linear scaling (shape factor $s = 1$) was applied in all cases, as no additional curvature was required.

The overall desirability was calculated as

$$D = d_{P,\mathrm{Fuco}}^{0.35} \cdot d_{P,\mathrm{EPA}}^{0.30} \cdot d_{P,\mathrm{Lipo}}^{0.15} \cdot d_{P,\mathrm{Hydro}}^{0.10} \cdot d_{\mathrm{P,Pellet}}^{0.06} \cdot d_{\mathrm{P,Emulsion}}^{0.04}$$

where each d_i denotes the individual desirability of the corresponding response. The weighting factors sum to unity and reflect the relative importance of each response. Fucoxanthin productivity (0.35) and EPA productivity (0.30) were prioritized as the principal value-driving objectives, followed by lipophilic (0.15) and hydrophilic (0.10) fraction productivities as co-product streams. Pellet fraction (0.06) and emulsion/interlayer (0.04) were included as penalty terms associated with residual solids and impaired phase separation, respectively. This weighting strategy was selected on a process knowledge basis, as scalable microalgal extraction processes must balance product recovery with process time, biomass loading, solvent efficiency, and downstream operability.

Based on the resulting desirability scores, the ten highest-ranking feasible conditions were selected as candidate operating points for further evaluation. The full equations, ranking criteria, and complete list of ranked candidate conditions are provided in the table above (Table S3.).
